# Diagnosis and treatment status of inoperable locally advanced breast cancer and the application value of inorganic nanomaterials

**DOI:** 10.1186/s12951-024-02644-9

**Published:** 2024-06-25

**Authors:** Linxuan Wu, Chuan He, Tingting Zhao, Tianqi Li, Hefeng Xu, Jian Wen, Xiaoqian Xu, Lin Gao

**Affiliations:** 1 Department of Ultrasound, Shengjing Hospital of China Medical University, Shenyang, 110022 China; 2 School of Intelligent Medicine, China Medical University, Shenyang, 110122 China; 3 Department of Laboratory Medicine, The First Hospital of China Medical University, Shenyang, 110001 China; 4 Department of Breast Surgery, The First Affiliated Hospital of China Medical University, Shenyang, 110001 China; 5 Department of Breast Surgery, The Fourth Affiliated Hospital of China Medical University, Shenyang, 110032 China

**Keywords:** Locally advanced breast cancer, Inorganic nanoparticles, Diagnosis, Local treatment

## Abstract

Locally advanced breast cancer (LABC) is a heterogeneous group of breast cancer that accounts for 10–30% of breast cancer cases. Despite the ongoing development of current treatment methods, LABC remains a severe and complex public health concern around the world, thus prompting the urgent requirement for innovative diagnosis and treatment strategies. The primary treatment challenges are inoperable clinical status and ineffective local control methods. With the rapid advancement of nanotechnology, inorganic nanoparticles (INPs) exhibit a potential application prospect in diagnosing and treating breast cancer. Due to the unique inherent characteristics of INPs, different functions can be performed via appropriate modifications and constructions, thus making them suitable for different imaging technology strategies and treatment schemes. INPs can improve the efficacy of conventional local radiotherapy treatment. In the face of inoperable LABC, INPs have proposed new local therapeutic methods and fostered the evolution of novel strategies such as photothermal and photodynamic therapy, magnetothermal therapy, sonodynamic therapy, and multifunctional inorganic nanoplatform. This article reviews the advances of INPs in local accurate imaging and breast cancer treatment and offers insights to overcome the existing clinical difficulties in LABC management.

**Graphical Abstract:**

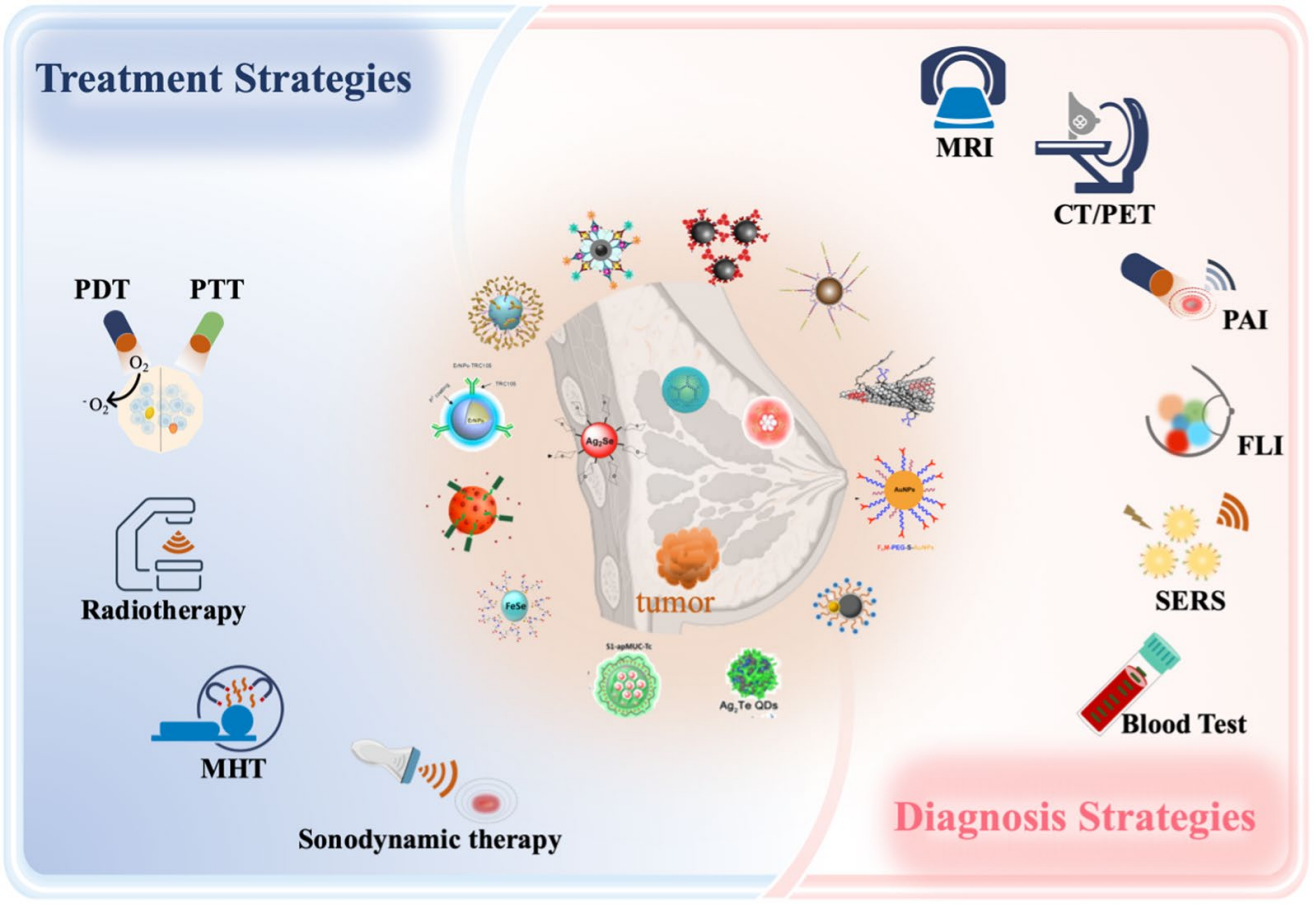

## Introduction

Locally advanced breast cancer (LABC) is a significant and complicated global public health concern. Currently, there are approximately 400,000–550,000 fresh cases of LABC diagnosed annually worldwide, accounting for 10–30% of all breast cancer cases [1–4]. In less-developed medical regions and several developing countries, the percentage of new breast cancer cases that are LABC can be as high as 40–60% [5]. LABC is defined as having a large tumor (> 5 cm, T3 according to AJCC 8th edition), a tumor of any size with involvement of the skin or chest wall (T4), a tumor with clinically detectable fixed regional axillary nodes (N2), or a tumor with ipsilateral infraclavicular, supraclavicular, or internal mammary lymph nodes (N3), excluding distant metastasis [6]. LABC can be divided into two types: operable LABC and inoperable LABC. Overall, LABC has a poor prognosis due to the presence of an enormous tumor burden, advanced stage, and a high probability of distant metastasis. The 5-year survival rate for LABC ranges from 48 to 52%, whereas the 10-year survival rate is less than 41% [7–9]. In particular, for inoperable LABC, the inability to surgically remove the tumor from the breast and regional lymph nodes allows tumor cells to infiltrate the lymphatic vessels in the skin easily. This event leads to regional and contralateral breast invasion, dramatically increasing the probability of hematogenous metastasis and ultimately leading to a worse prognosis. Systemic treatment is the primary treatment for inoperable LABC [10]. Without timely and effective systemic treatment, patients often suffer from complications caused by local tumor invasion, such as ulceration, bleeding, odor, pain, and severe upper limb edema. An even bigger concern is that these patients often experience distant metastasis within a short period, which can inevitably result in death. Neoadjuvant therapy before surgery assists in reducing the stage of inoperable LABC, providing the opportunity to undergo radical surgical treatment. However, patients who do not respond well to current neoadjuvant therapy regimens are unable to undergo radical surgery. Instead, they can only receive palliative systemic treatment and radiotherapy. This inability to effectively control disease progression significantly increases the likelihood of distant metastasis and results in a poor prognosis.

The field of inorganic nanoparticles (INPs) has witnessed significant progress, leading to the emergence of innovative approaches and strategies for diagnosing and treating breast cancer [11]. These methods capitalize on the specific biological characteristics of inorganic nanomaterials, which have shown tremendous potential for translational clinical applications, particularly in addressing the treatment restrictions of inoperable LABC. This article first introduces the current diagnostic and treatment pathways and challenges for inoperable LABC. This paper primarily focuses on the new technological applications of nanomaterials in the precise imaging assessment of breast cancer, radiosensitization, novel local treatment approaches, and the applications of diagnostic and therapeutic studies. This study aims to provide a comprehensive review of the diagnosis and treatment of inoperable LABC from the perspective of INPs, as well as offer fresh insights into the translational clinical applications of these INPs.

## Current treatment protocol and challenges in the management of inoperable LABC

**Fig. 1:**
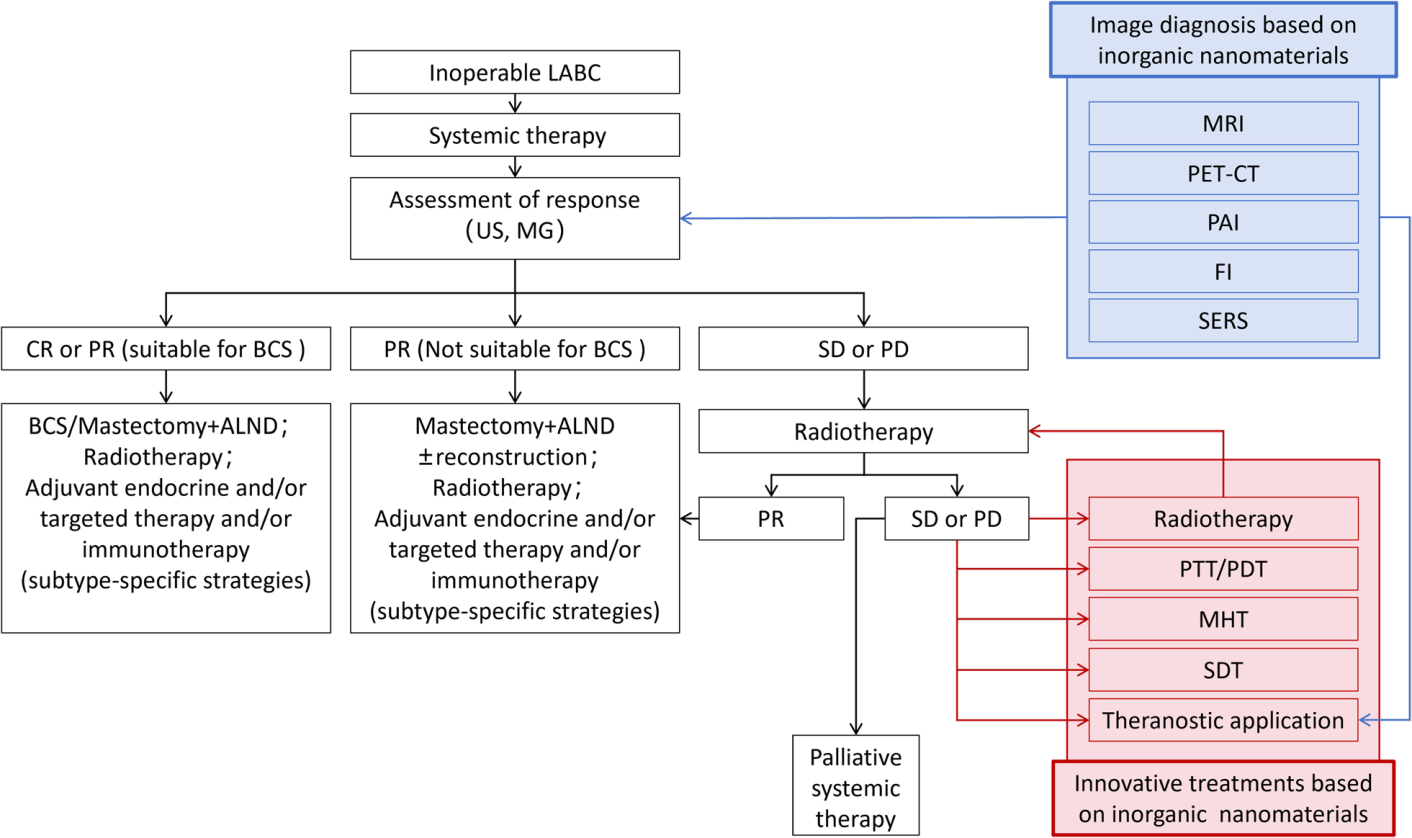
Treatment of locally inoperable breast cancer and application prospects of INPs. *US* ultrasound, *MG* mammography, *MRI* magnetic resonance imaging, *PET-CT* positron emission tomography-computed tomography, *PAI* photoacoustic imaging, *FI* fluorescence imaging, *SERS* surface-enhanced Raman scattering, *CR* complete response, *PR* partial response, *SD* stable disease, *PD* progressive disease, *BCS* breast-conserving surgery, *ALND* axillary lymph node dissection, *PTT* photothermal therapy, *PDT* photodynamic therapy, *MHT* magnetic hyperthermia therapy, *SDT* sonodynamic therapy

Presently, the treatment options for inoperable LABC include local treatments such as surgery and radiotherapy and systemic treatments like chemotherapy, targeted therapy, endocrine therapy, and immunotherapy [6]. Systemic treatment remains the primary approach, supplemented by local treatment due to the significant burden of tumors, extensive invasion, and the high risk of distant metastasis. The selection of systemic treatment regimens needs to be tailored to the subtype of breast cancer. Most patients with inoperable LABC inevitably must undergo chemotherapy. Endocrine therapy is used in hormone receptor-positive (ER positive and PR positive) breast cancer, whereas HER2-positive breast cancer requires anti-HER2 therapy [12]. Optimizing the diagnosis and treatment process for inoperable LABC is essential to enhance treatment outcomes. Compared to the traditional approach of adjuvant systemic treatment plus radiotherapy after surgery, the value of neoadjuvant therapy conducted before surgery is increasingly recognized. It is gradually becoming the widely accepted standard treatment for inoperable LABC [4]. Effective neoadjuvant therapy can reduce the stage of inoperable LABC, allowing patients to downstage to radical surgery and receive subsequent intensified adjuvant treatment. This approach has significantly reduced the recurrence and mortality rates of patients with inoperable LABC [7, 13]. Based on the emergence of additional substantiation from evidence-based medicine, the current treatment protocol for inoperable breast cancer is described as follows (Fig. 1).

For patients with inoperable LABC, accurate imaging assessments and pathological diagnosis are crucial in determining the extent of tumor invasion, pathological features, and tumor subtypes. Subsequent treatments involve personalized, subtype-specific neoadjuvant therapies and whole-course imaging monitoring to assess changes in tumor burden and the efficacy of the neoadjuvant therapies according to Response Evaluation Criteria in Solid Tumors (RECIST). The RECIST assessment categorizes tumor response post-treatment as CR, PR, SD, or PD [14]. Patients who exhibit a positive response to treatment, with a significant decrease in the tumor size or even complete response (PR/CR), may qualify for BCS or mastectomy. The following treatments include radiotherapy and subtype-specific adjuvant therapies such as endocrine therapy, targeted therapy, and immunotherapy [10, 12]. Patients who have a relatively effective treatment response but are not suitable for BCS may undergo mastectomy, with or without breast reconstruction. Patients with a poor treatment response, assessed as SD or PD, require local radiotherapy to become eligible for radical surgery and receive subsequent adjuvant therapy. If radiotherapy is unfortunately ineffective, these patients have to enter the palliative systemic treatment stage, which often carries the worst prognosis [15].

The treatment process mentioned above for inoperable LABC emphasizes the importance of achieving the opportunity for radical surgery via effective neoadjuvant therapy as the initial crucial step in treatment, which is necessary for subsequent adjuvant therapy. With the continuous emergence of new treatment drugs such as CDK4/6 inhibitors, immunotherapy, and antibody–drug conjugates, the selection of neoadjuvant therapy regimens has become more diverse and effective. This outcome has further increased the possibility of transforming non-surgical LABC after neoadjuvant therapy into surgical LABC, providing the possibility for radical surgery [10, 12].

Another key point for treating inoperable LABC is precise imaging monitoring and assessment. Accurate imaging diagnosis is crucial for making appropriate clinical treatment decisions. A precise imaging assessment of treatment response is essential for accurately determining the viability of radical surgery. Imaging information is a critical reference in clearly identifying surgical resection margins, selecting appropriate surgical procedures, and delineating the target area for local radiotherapy [16]. Conventional breast US and MG are not precise enough to confirm the extent of tumor invasion in inoperable LABC, which often exhibits a widespread distribution. Breast-enhanced MRI provides higher imaging accuracy for assessing tumor invasion and is more suitable for evaluating treatment responses [17, 18]. PET-CT can provide more sensitive monitoring data, especially for detecting distant metastases [19, 20]. However, for inoperable LABC with extensive regional invasion, the current imaging methods mentioned above are still unable to meet the demands for the complete determination of tumor boundary information due to their limited resolution.

The third key point in treating inoperable LABC is practical local therapy. For cases where neoadjuvant therapy is ineffective and surgery is not feasible, radiotherapy is currently the only viable local treatment option. However, the limited tolerance of body tissues to radiotherapy dramatically hinders the ability to increase radiation dosage. High-dose radiotherapy can result in various complications, such as skin fibrosis, skin breakdown, lung fibrosis, myocardial damage, rib necrosis, brachial plexus injury, and severe edema in the affected upper limb [21]. There is an urgent need for the development and clinical applications of novel treatment methods, in addition to radiotherapy, for local treatment to effectively improve the chances of converting patients with non-surgical LABC into candidates for surgery, enabling them to undergo radical surgical procedures. This move will also provide better local control treatment for patients relying on palliative systemic therapy, ultimately improving their quality of life [22].

## New solutions for inoperable LABC using INPs

Based on the background above, the diagnostic and treatment challenges related to inoperable LABC must be tackled as soon as possible. This option includes developing and applying novel therapeutic drugs, facilitating advancements in imaging techniques, and exploring and implementing innovative local treatment methods. Systemic treatment drugs for breast cancer have improved significantly in recent years, and numerous reviews have summarized these advancements. This article does not delve into that realm. Instead, it summarizes and analyzes the literature on breast cancer imaging and local treatment.

First, bibliometric analysis was employed to measure the impact of research articles, assist researchers in identifying future research trends, and focus on critical areas of study. This study’s authors have conducted a comprehensive bibliometric analysis of the literature related to the applications of nanomaterials in breast cancer over recent decades. This analysis evaluates the research trends and hot topics in diagnosing and treating breast cancer using nanomaterials from a bibliometric perspective.

**Fig. 2:**
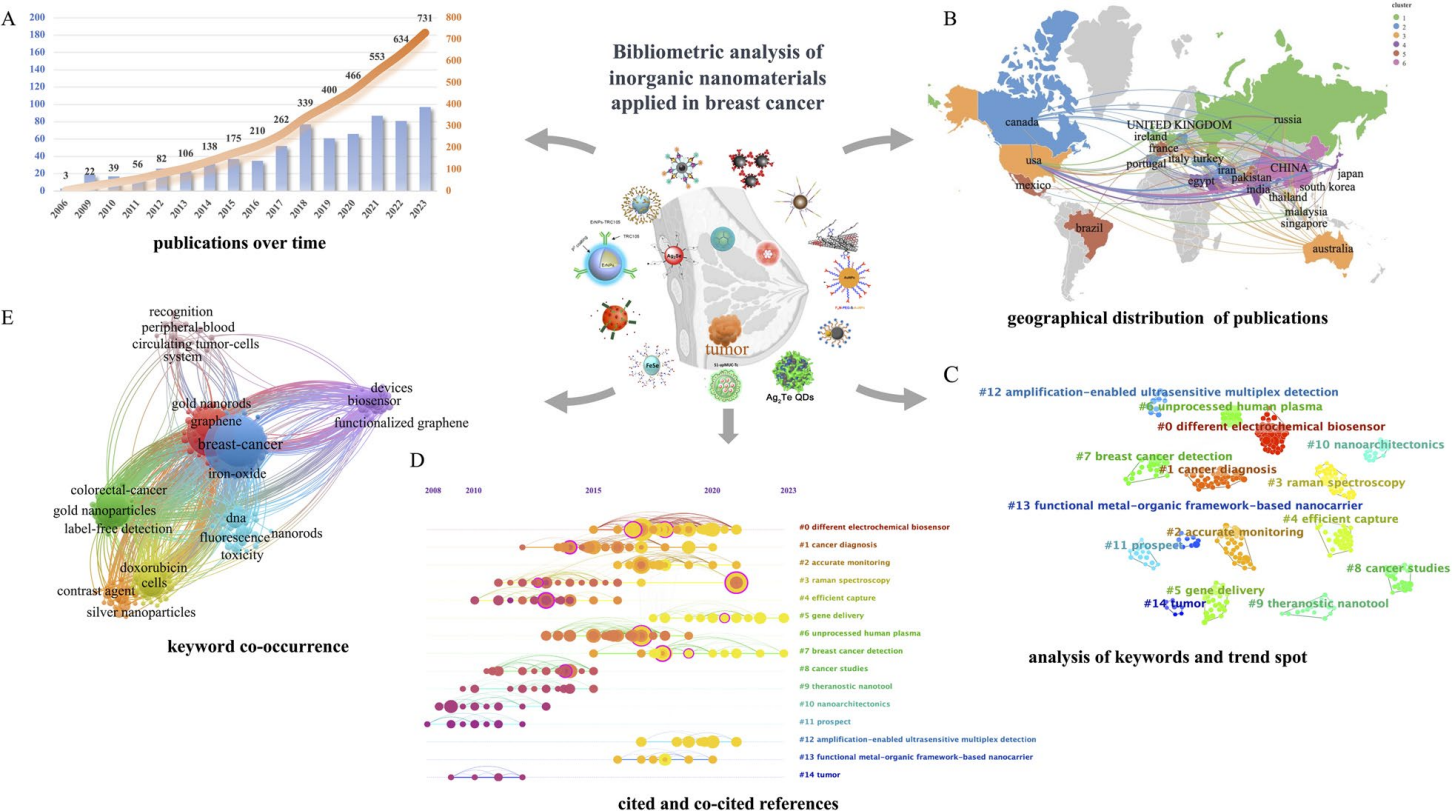
Bibliometric analysis of INPs applied in breast cancer. **A** Annual and cumulative trend of publication from 2006 to 2023; **B** world map of global overview and international collaboration; **C** 15 clusters of key hotspots divided by CiteSpace software. Distinct clusters are color-coded for identification; **D** a timeline visualization of 15 clusters generated by CiteSpace software; **E** keyword co-occurrence analysis map produced by VOSviewer and Pajek. Nanoparticle’’s image is adapted with permission from Ref. [33–47], copyright 2019 Journal of Materials Chemistry B, 2020 International Journal of Nanomedicine, 2022 Proceedings of The National Academy of Sciences of the United States of America, 2022 International Journal of Molecular Sciences, 2019 Science Advances, 2017 Nanomedicine, 2020 Acs Applied Materials & Interfaces, 2020 Talanta, 2021 Analytical And Bioanalytical Chemistry, 2018 Science Advances, 2019 Nano Letters, 2018 Acta Biomaterialia, 2020 Journal of Materials Chemistry B; 2018 Theranostics, 2023 Angewandte Chemie-International Edition

This study incorporated 755 records that met the search criteria. Figure 2A uses a blue bar to depict the temporal distribution of publications related to the applications of INPs in diagnosing and treating breast cancer. The orange curve illustrates the cumulative total number of publications over the years. From 2006 to 2023, and especially since 2017, the number of annual publications has shown an uptrend, indicating that the application of INPs in breast cancer is emerging as a research hotspot.

Next, the authors analyzed the academic cooperation and exchanges between countries and institutions in this field from 2006 to 2023 and discovered the participation of researchers from 29 countries and regions. Figure 2B provides a global overview of these studies. This study conducted a cluster analysis of the quantity of international collaborative publications and classified these international relationships into six distinct clusters. The thickness of the lines indicates the frequency of collaboration between institutions. Notably, from 2006 to 2023, China and the United States exhibited the closest cooperation, jointly publishing 21 articles.

The researchers next utilized an overlay of network and density visualizations to analyze the keywords and their co-occurrence of nanomaterials in breast cancer research. The top 15 clusters of key hotspots were identified by analyzing 2098 keywords. The three clusters with the most contributions included “different electrochemical biosensors,” “cancer diagnosis,” and “accurate monitoring” (Fig. 2C). A timeline-based analysis was conducted to understand how these clusters were distributed across various periods (Fig. 2D). An evolution of researching themes over time was observed. Early references emphasized nanoarchitectonics and theranostics nanotools, whereas recent publications focused on gene delivery and breast cancer detection employed with nanomaterials.

Keyword co-occurrence analysis revealed that the research hotspots could be divided into eight categories (Fig. 2E). The blue cluster primarily encompassed keywords related to breast cancer, whereas the green cluster was primarily associated with gold nanoparticles (AuNPs). The keywords co-occurring with breast cancer are closely linked to INPs such as iron oxide, gold, silver, and graphene. These keywords also strongly connect to diagnostic techniques like circulating tumor cells (CTC), biosensors, fluorescence, and contrast agents.

The bibliometric analysis results clearly demonstrate that the value of INPs applied in breast cancer research is gradually being recognized and emphasized by researchers worldwide. The development and applications of INPs, as one of the hotspots in cancer therapy, offer innovative ideas and solutions for advancing imaging techniques [23]. The development and applications of novel inorganic nanomaterial contrast agents have significantly enhanced the anatomical resolution of CT, MRI, and PET-CT [24, 25]. Furthermore, the development of novel imaging techniques, such as PAI, FI, and SERS, based on inorganic nanomaterial technology, has significantly improved the accuracy of tumor imaging assessment [26–28].

In the field of local therapy, INPs have introduced innovative treatment possibilities in the areas of radiotherapy sensitization, PTT, PDT, MHT, and SDT [29, 30]. These advancements show promising potential for clinical applications. The potential to perform imaging, diagnosis, and treatment simultaneously, known as theranostics, using INPs is exciting. It presents significant opportunities for theranostics applications in the local treatment of inoperable LABC [31, 32]. The following text will provide a detailed introduction to the progress of translational research on inorganic nanomaterials for their potential clinical applications in diagnosing and treating inoperable LABC.

## INPs for the diagnosis of LABC

The bibliometric analysis shows that INPs have made breakthroughs in diagnosing breast cancer by enhancing the signal intensity of various optical imaging to increase sensitivity and specificity, especially the development of multimodal imaging technology that facilitates early-stage diagnosis to guide personalized and precision treatment.

Breast cancer is a complex disease characterized by the development of malignant tumors in breast tissues, often manifesting as lumps and changes in the breast shape or texture. Its progression varies widely based on molecular tumor subtypes, such as hormone receptor status, the timing of therapeutic intervention, and individual patient factors.

Based on the 2015 St. Gallen early breast cancer international expert consensus [48], breast cancer can be categorized into four main types by the expression of specific biomarkers: (1) Luminal A: this type is hormone receptor-positive (ER positive and PR positive) and HER-2 negative. It is one of the most common forms of breast cancer, accounting for about 60% of all cases. (2) Luminal B: this type is hormone receptor-positive and can be either HER-2 positive or negative, representing approximately 20–30% of breast cancer cases. (3) HER-2 positive: this type is characterized by being hormone receptor-negative and accounts for about 10–20% of breast cancer cases. It generally has a relatively poor prognosis. (4) Triple-negative breast cancer (TNBC): this type lacks both hormone receptors and the HER-2 receptor, comprising roughly 10% of breast cancer cases. TNBC typically has a poorer prognosis, is unresponsive to endocrine therapy and targeted treatments, and offers fewer therapeutic options.

Data from the National Breast Cancer Foundation (https://www.nationalbreastcancer.org/breast-cancer-facts/) indicate that when breast cancer is detected early at the localized stage, the 5-year relative survival rate can be as high as 99%. This finding underscores the significant benefit of early detection in improving survival outcomes for patients with breast cancer.

MG, an X-ray test of the breast, is a primary method for breast cancer screening and diagnosis [49]. However, X-ray image interpretation relies on the experience and subjective judgment of radiologists, leading to potential misdiagnoses or missed diagnoses. Furthermore, mammograms may lack sufficient sensitivity for the early detection of breast cancer, especially in dense breast tissues.

Given these limitations, multiple imageological examinations, including MRI, CT, PET, PAI, SERS, and FLI, can be applied for the early detection of breast cancer. INPs are superior sensitive probe materials due to their unique acoustic, electrical, optical, magnetic, and thermal properties. In recent years, numerous INPs have been used to provide higher-resolution images to detect early breast cancer lesions (Table 1).

**Table 1:** Summary of breast cancer diagnosis based on inorganic nanoplatforms

Inorganic nanoplatform		Size (nm)	Cell type	Cell safety concentration (incubation time)	Animal tumor model	Application(s)	Ref.
Fe	SPIONs	~ 20	4T1	100 μg/mL (24 h)	Orthotopic 4T1 mice model	MRI	[50]
	Fe_3_O_4_ Jnps	~ 51	4T1	1000 μg/mL (24 h)	Subcutaneous 4T1 mice model	MRI	[51]
	MFNPs	18	4T1	None	Orthotopic 4T1 mice model	MRI	[43]
	FeSe QDs	3.4 ± 0.3	MCF-7	None	Subcutaneous MCF-7 mice model	FLI	[37]
Gd	APT-QD-NPs	2–3	4T1	None	Subcutaneous 4T1 mice model	MRI/FLI	[52]
	TUG	82	4T1	100 μg/mL (48 h)	Subcutaneous 4T1 mice model/orthotopic 4T1 mice model	MRI	[53]
Ag	Ag_2_Te QDs	4.3	4T1	200 ppm (24 h)	Subcutaneous 4T1 mice model	CT	[39]
	Ag_2_Te NPs	17.5	MDA-MB-231	1 mg Ag/mL (4 h)	Orthotopic MDA-MB-231 mice model	CT	[54]
	Glc-Ag_2_Se QDs	2.4 ± 0.5	MCF-7	1 g/mL (72 h)	Subcutaneous MCF-7 mice model	FLI	[33]
	PPA@AgNDs	6–7	4T1	100 μg/mL (48 h)	Subcutaneous 4T1 mice model	FLI	[34]
Au	PEG-S-AuNPs	3.7 ± 0.6	MDA-MB-231	10–25 μg/mL (24 h)	Subcutaneous MDA-MB-231 mice model	CT	[36]
	GNR-MS-FA	90 ± 10	4T1	1 mg/mL (24 h)	Subcutaneous 4T1 mice model	CT	[55]
	AuFe_3_O_4_@PDA-PEG-DTPA-Gd	32 ± 3	MDA-MB-231	100 mg Gd/L (24 h)	Orthotopic MDA-MB-231 mice mode	MRI/CT	[45]
	mPEG@HGNPs	46.3 ± 0.166	MCF-7	None	Subcutaneous MCF-7 mice model	CT	[56]
	AuNPs	22.51–27.36	MCF-7/4T1	100 μg/mL (72 h)	Orthotopic 4T1 mice model	PAI	[57]
	AuNPs	25	4T1	50 μg/mL (96 h)	Orthotopic 4T1 mice model	PAI	[58]
	J-ACP	85	4T1	None	Subcutaneous 4T1 mice mode	PAI	[59]
	DNCs	276.90 ± 110.50	4T1	200 μg/mL (24 h)	Orthotopic 4T1 mice model	US	[60]
	Pt-HAuNS-PFH@O_2_	~ 50	MDA-MB-231	None	Subcutaneous MDA-MB-231 mice mode	US	[61]
	DTNB-AuNS /pMBA-AuNS	50–70	MDA-MB-231	None	Orthotopic MDA-MB-231 mice mode	SERS	[62]
	HAuNP@DTTC	70–85	4T1	None	Orthotopic 4T1 mice model	SERS	[63]
Pt	Fu-PtNPs	33 ± 3.4	MCF-7	3 μg/mL	Subcutaneous MCF-7 mice model	CT	[64]
Si	MSNs	114	MDA-MB-231	200 μg/mL (48 h)	Subcutaneous MDA-MB-231 mice model	SPECT	[38]
Ga	DOTA–BN–TMC–MNPs	20–30	T-47D	200 μg/mL (24 h)	Subcutaneous T-47D mice model	PET/MRI	[65]
C	CQD-KD1	5–8	4T1/MCF-7	100 μg/mL (24 h)	Subcutaneous 4T1 mice mode	FLI	[66]

SPIONs: superparamagnetic iron oxide nanoparticles; Jnps: Janus nanoparticles; MFNPs: magnetic ferrite nanoparticles; QDs: quantum dots; APT: aptamer; TUG: Tf-USPIO@Gd(III); Glc: glucose; PPA: porcine pancreatic α-amylase; PEG: polyethylene glycol; GNR: gold nanorods; PDA: polydopamine; DTPA: diethylenetriaminepentaacetic acid; mPEG@HGNPs: PEGylated hollow gold nanoparticles; DNCs: dual-targeted gold nanoshelled poly(lactide-co-glycolic acid) nanocapsules carrying vascular endothelial growth factor receptor type 2 (VEGFR2) and p53 antibodies; Pt-HAuNS-PFH@O_2_: oxygen-saturated perfluorohexane-cored, cisplatin (Pt)-decorated hollow gold nanospheres; DTNB: 5,5′-dithiobis(2-nitrobenzoic acid); pMBA: 4-mercaptobenzoic acid; DTTC: 3.3-diethylthiatricarbocyanine iodide; Fu-PtNPs: fucoidan-coated Pt nanoparticles; MSNs: mesoporous silica nanoparticles; DOTA–BN–TMC–MNPs: *N*,*N*,*N*-trimethyl chitosan (TMC)-coated magnetic nanoparticles (MNPs) conjugated to *S*-2-(4-isothiocyanatobenzyl)-1,4,7,10-tetraazacyclododecane tetraacetic acid (DOTA) as a radioisotope chelator and bombesin (BN) as a targeting peptide; CQD-KD1: kunitz domain 1 with carbon quantum dots

The following section will focus on the application and development of INPs in assisting these breast cancer diagnosis techniques.

### Magnetic resonance imaging

MRI utilizes powerful magnetic fields and radio waves to generate detailed images of internal structures and organs within the body, thus enabling the identification of malignant breast lesions by analyzing their morphological and dynamic characteristics. Nevertheless, the applications of MRI in breast cancer diagnosis are hindered by certain limitations, such as inadequate spatial resolution and possible toxic side effects associated with conventional contrast agents like gallium [67, 68]. The application of INPs is a promising solution to overcome these drawbacks. In recent 5 years, it has been widely reported that INPs [69] with iron (Fe), gadolinium (Gd), cobalt (Co), nickel (Ni) manganese (Mn), dysprosium (Dy), holmium (Ho), ferrites (various compositions) and magnetite (Fe_3_O_4_) have been used as magnetic sensitizers for breast cancer, especially LABC diagnosis, which can enhance MRI signal intensity and resolution. These INPs enhance the contrast between tumors and surrounding normal tissues, easing the detection and localization of tumors.

**Fig. 3:**
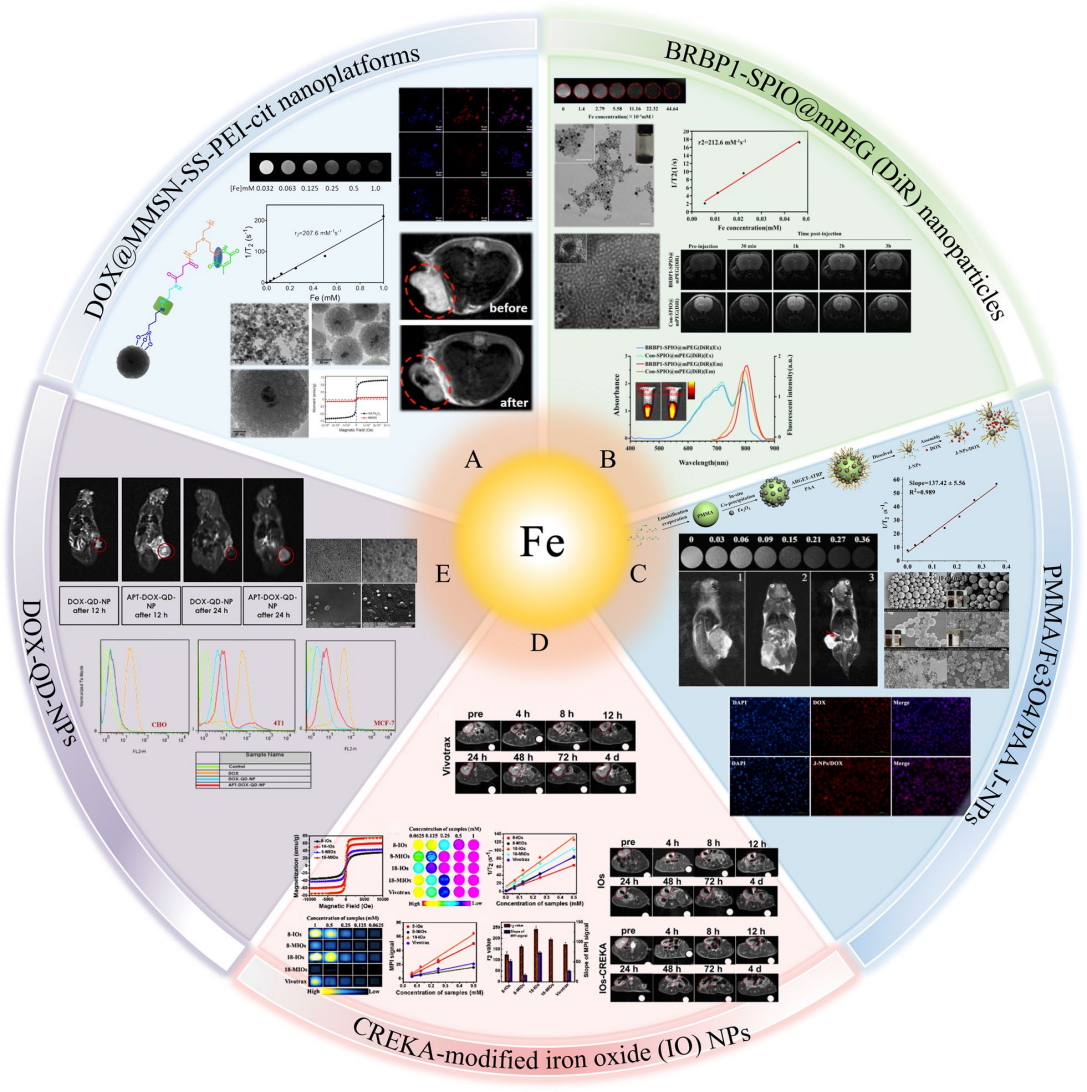
A summary of SPIONs available for MRI in breast cancer diagnosis. **A** DOX@MMSN-SS-PEI-cit nanoplatforms; **B** BRBP1-SPIO@mPEG (DiR) nanoparticles; **C** PMMA/Fe3O4/PAA Jnps; D. CREKA-modified iron oxide (IO) NPs; **E** DOX-QD-NPs. **A** Is adapted with permission from [50], copyright 2020 Journal of Colloid and Interface Science. **B** Is adapted with permission from [72], copyright 2020 Materials Science and Engineering: C. **C** Is adapted with permission from [51], copyright 2020 Biomaterials Science. **D** Is adapted with permission from [43], copyright 2019 Nano letters. **E** Is adapted with permission from [52], copyright 2020 International Journal of Pharmaceutics

**Fig. 4:**
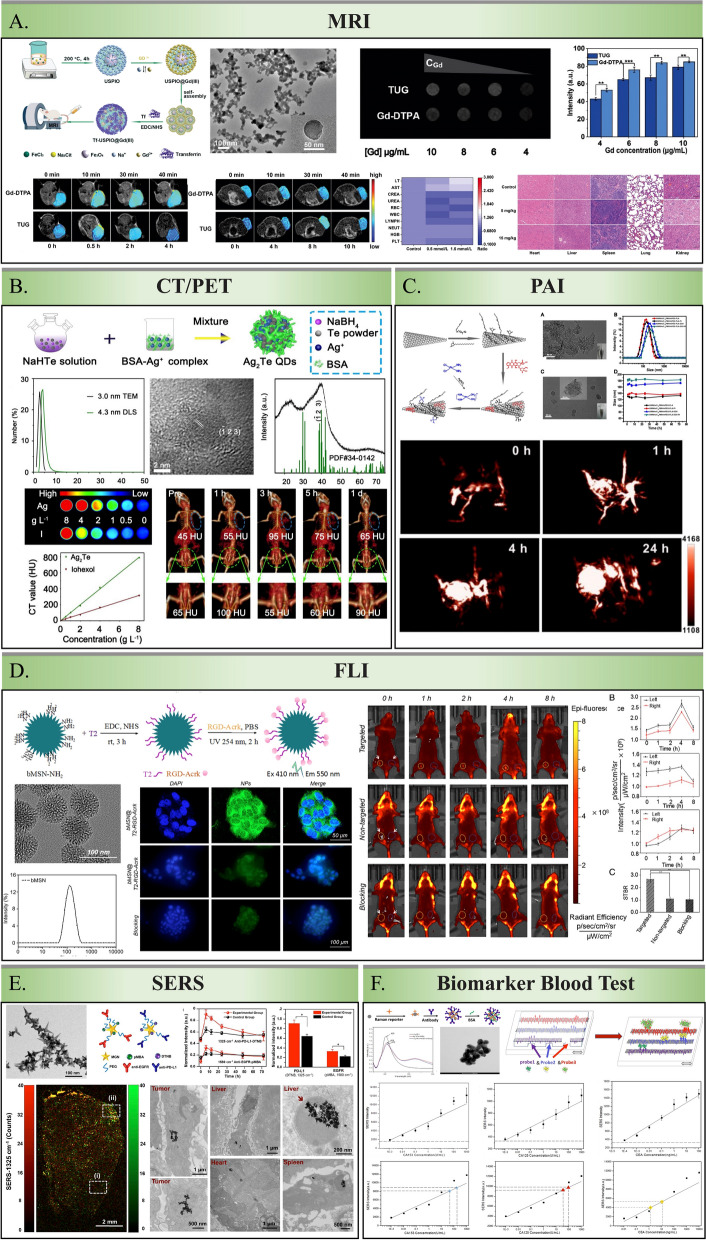
INPs for the diagnosis of breast cancer. **A** TUG; **B** Ag_2_TeQDs; **C** SWNHs/C18PMH/mPEG-PLA-DOX-Pt; **D** bMSN@T2-RGD-Acrk; **E** AuNS; **F** biosensor that combined CA153, CA125 and CEA antibodies with DTNB, 4MBA, and 2NAT labeled Ag nanomaterials. **A** Is adapted with permission from [53], copyright 2020 Journal of Materials Chemistry B. **B** Is adapted with permission from [39], copyright 2020 ACS Applied Materials & Interfaces. **C** Is adapted with permission from [46], copyright 2018 Theranostics. **D** Is adapted with permission from [86], copyright 2019 Nanomedicine. **E** Is adapted with permission from [62], copyright 2018 nanoscale. **F** Is adapted with permission from [92], copyright 2018 Talanta

SPIONs have been extensively investigated as MRI contrast agents due to their low toxicity and preferable biocompatibility. Their target-specific properties enable them to selectively bind to breast cancer cells; moreover, heightened sensitivity enables them to detect subtle changes in breast lesions early. Figure 3 summarizes SPIONs currently available for MRI in breast cancer. Zhou et al. [53] constructed a transferrin-modified gadolinium-iron chelate nanoprobe based on ultra small paramagnetic iron oxide nanoparticles (USPION) named TUG. The obtained TUG demonstrated high biocompatibility even at a high dose of 15 mg kg^−1^. More importantly, compared with clinically used Gd-based small molecule contrast agents, TUG can be more engulfed by 4T1 cells, showing much enhanced T_1_-weighted positive MRI in both subcutaneous and orthotopic tumor models of breast cancer (the highest signal value of Magnevist = 390, whereas TUG = 430) (Fig. 4A).

T_2_ contrast agents are typically superior to conventional T_1_ agents in MRI imaging primarily due to their higher relaxivity ratios (r_2_/r_1_). This elevated r_2_/r_1_ ratio indicates that contrast agents are more effective in enhancing image contrast, demonstrating a more pronounced performance advantage in MRI imaging [70]. Li et al. [71] conducted SPIO/DSPE-PEG5k-(Bom&Cy5) nanomicelles for dual-modality MR/near-infrared (NIR)/FI imaging in MDA-MB-231 breast cancer cells. The nanomicelles exhibited a high transverse relaxivity (r_2_) of 493.9 mM^−1^ s^−1^, revealing a linear dependence on Fe concentration and indicating their effectiveness as T_2_ contrast agents in MRI. Furthermore, in vitro targeting efficiency results revealed that Bom-targeted nanomicelles have a strong affinity toward GRPR-positive cells, suggesting their potential as active targeting contrast agents for precise imaging in breast cancer diagnostics. Ran et al. [50] reported a novel type of monodisperses mesoporous silica-coated superparamagnetic iron oxide-based multifunctional nanoplatform (DOX@MMSN-SS-PEI-cit) with a high r_2_ value of 207.6 mM^−1^ s^−1^. T_2_-weighted MRI images of 4T1 tumor-bearing mice were captured before and after the tail vein injection of DOX@MMSN-SS-PEI-cit. They observed a significantly decreased signal in the region of the breast tumor in post-injection mice, indicating that the DOX@MMSN-SS-PEI-cit nanoplatform has tumor-targeting properties and acts effectively as an MRI contrast agent while enhancing the diagnostic capability of MRI.

In addition to iron nanomaterials, Mona Alibolandi et al. [52] synthesized Gd-doped copper indium zinc sulfide QDs attached with AS1411 DNA Apt (a single strand DNA Apt with high affinity against nucleolin, which is overexpressed at the cell surface as breast cancer marker) for diagnosing breast cancer. They injected the nanomaterial into 4T1 tumor-bearing Balb/c mice and acquired T_2_-weighted MR images 12 and 24 h after injection. Compared with the untargeted agent, the targeted agent linked to AS1411-Apt significantly accumulated at the tumor site, as demonstrated by MRI, highlighting the platform’s capability for enhanced tumor targeting and imaging.

Using INPs as magnetic sensitizers in MRI provides advanced capabilities for breast cancer detection and precise targeting. This approach significantly enhances diagnostic efficiency, enabling the early-stage identification and monitoring of disease progression.

### Computed tomography and positron emission tomography

Molecular imaging modalities, including PET and CT, have been evaluated for primary breast cancer diagnosis and staging in recent years. These two imaging techniques use nuclides to distinguish normal tissue from diseased tissues, especially for diagnosing tumors [73, 74].

Currently, nuclides used in PET and CT contrast enhancement include Gallium-68, FDG, Carbon-11, Nitrogen-13, and Oxygen-15. These isotopes have short half-lives, which imposes strict limitations on their production, transportation, and usage windows. INPs overcome these drawbacks by avoiding radiation exposure issues as novel imaging agents. Flexible size and composition, high biocompatibility, and reduced toxicity and immune responses make them suitable for multimodal imaging.

Shen et al. synthesized mPEG@HGNPs, and the attenuation properties were examined by CT imaging in MCF7 breast cancer cells [56]. Compared to HGNPs and iohexol, mPEG@HGNPs demonstrated enhanced CT attenuation intensity and brightness. In xenografted tumor-bearing BALB/c mice, mPEG@HGNPs maintained contrast enhancement at the tumor site for 12 h post-injection (ΔHu 89), outperforming HGNPs (ΔHu 73 at 4 h, ΔHu 10 at 12 h), suggesting prolonged blood half-life and targeted organ accumulation due to reduced uptake by the mononuclear phagocyte system.

Recently, researchers have focused on inorganic QDs to improve their performance in biomedical imaging, cell labeling, in vivo imaging, and other multimodal imaging techniques instead of traditional CT or PET imaging. Hong et al. observed that Ag_2_Te QDs exhibited a remarkably enhanced contrast effect of CT imaging in a concentration-dependent manner in vitro experiment [39]. At the equivalent concentration, Ag_2_Te QDs possessed a Hounsfield units value of 99 HU L g^–1^. At the same time, iohexol, a commonly used clinical contrast agent, exhibited a value of only 39 HU L g^–1^, demonstrating the potential high performance of Ag_2_Te QDs for contrast-enhanced CT imaging. Furthermore, the CT imaging signal of the breast tumor tissue was more than twice as strong at 3 h after injection compared with the pre-contrast image (Fig. 4B). These results indicated that enhanced CT imaging quality provided by Ag_2_Te QDs could result in earlier and more accurate diagnoses.

CT imaging accurately provides anatomical and pathological information. Combined with PET imaging, which offers functional and metabolic insights, this dual-modality approach is highly beneficial for a comprehensive assessment of breast cancer. Furthermore, INPs significantly enhance such dual-modality detection of breast cancer, leveraging the strengths of both CT and PET for improved early diagnosis.

Beiki et al. used ^68^Ga–DOTA–BN–TMC–MNPs (SPIONs-based) nanomaterials to perform PET and CT dual-modality imaging of nude mice subcutaneously implanted with T-47D cells [65]. Enhanced tumor visibility was observed in PET imaging even at a lower nanoparticle concentration of 0.62 mg/mL due to its high sensitivity. Besides, the SUVmax (standardized uptake value) ratio and SUVpeak ratio of the tumor vs control group were calculated using PET/CT scans at 120 min and obtained as 19.6 and 15.4, respectively. These results indicated that the high uptake of this nanoparticle in the tumor lesions could allow for precise tumor imaging for an early diagnosis of breast cancer.

The application of INPs to CT and PET imaging has significantly revolutionized the diagnosis efficiency of breast cancer. By providing enhanced contrast and detailed dual-modality imaging, this innovation enhances diagnostic precision and supports more sensitive monitoring of disease progression.

### Photoacoustic imaging

PAI, an emergent non-invasive imaging technique, combines the benefits of ultrasonic imaging with greater flexibility in the selection of photosensitizers (PSs) [75]. The images of internal tissues are produced according to the photoacoustic effect. This method involves directing short laser pulses at tissues, which absorb the light and rapidly expand thermally, generating acoustic waves. These waves are detected by ultrasonic sensors, and the data are used to create detailed images that reflect the tissues’ optical absorption characteristics, which are crucial for diagnosing cancer [76]. The effect of imageological examinations is affected mainly by tissue thickness. Despite the variations in breast tissue thickness among individuals, typically ranging from a few centimeters to tens of centimeters, PAI’s penetration depth of 7 cm is adequate to cover most breast tissues. Along with its spatial resolution of 100 µm, PAI enables the detection of subtle changes associated with early-stage breast cancer, thus significantly enhancing the accuracy of early diagnosis and distinguishing tumor types and progression stages [77, 78]. PAI primarily generates images based on the light absorption of biological tissues. However, benign and malignant tumors exhibit similarities in specific physiological and biochemical characteristics, such as blood vessel density, oxygenation levels, and metabolic activity [79, 80]. Consequently, PAI may pose challenges in distinguishing malignant tumors from benign tumors. Moreover, image quality largely depends on the stability of PAI signals. Researchers have turned to INPs for targeted and stable imaging to address these limitations.

Recently, gold, antimony, bismuth, cobalt, copper, palladium, silver, titanium, tungsten, uranium, carbon nanomaterials, and graphene have been utilized as imaging elements in PAI to provide high-contrast photoacoustic signals that significantly enhance the accuracy and efficiency of breast cancer diagnosis [69].

Ran et al. synthesized CuS@mSiO_2_-PFP-PEG (CPPs) nanocomposites and found them highly effective as a contrast agent for PAI [81]. Their study observed that the photoacoustic signal intensity linearly increased with CPP concentrations ranging from 100 to 1500 μg/mL. Furthermore, they conducted experiments on tumor-bearing mice models, where they acquired PAI of tumor sites at various time intervals (0, 3, 6, 12, 24, and 48 h) following the intravenous injection of CPPs. They discovered that the CPPs increased the PA signal intensity of the tumor regions from 0.073 to 0.161 within 24 h post-injection, demonstrating the accumulation of CPPs in the tumor sites. These results highlight the potential of CPPs in enhancing the PAI of tumors, aiding in more accurate and effective diagnosis.

Zhang et al. used single-walled carbon nanohorns (SWNHs) as contrast agents for PAI and synthesized SWNHs/C18PMH/mPEG-PLA-DOX-Pt [46]. They focused on its potent PAI characteristics for effective tumor targeting and accumulation. PAI analysis revealed that after intravenous administration at 10 mg/kg in 4T1 tumor-bearing mice, the nanohorns progressively accumulated in the tumor. Notably, significant accumulation was observed at 24 h post-injection in both tumor vessels and parenchyma. These characters underscore the nanohorns’ ability to persist in the tumor environment, enabling clear and detailed imaging crucial for precise tumor diagnosis (Fig. 4C).

Xu et al. conducted Janus-structured chitosan/gold nanohybrids (J-ACP) for PAI-guided PTT in breast cancer [59]. They administered 100-μL J-ACP (5.23 mg/mL) intravenously to 4T1 tumor-bearing mice and recorded PA signal intensity at different time points. Their results demonstrated J-ACP actively accumulating at tumor sites and significantly enhanced PAI signals, emphasizing its potential in breast cancer diagnostics.

INPs utilized as imaging elements in PAI significantly boost breast cancer's detection capabilities [82] and improve the dynamic observation and diagnosis precision of tumors by providing high-contrast signals.

### Fluorescence imaging

FLI is a technique that utilizes the fluorescent properties of substances to emit light at specific wavelengths. FLI is characterized by real-time detection and high sensitivity, enabling early detection of breast cancer cells and small metastases. However, in breast cancer diagnosis, FLI requires highly selective and stable fluorescent probes, whereas unstable or non-specific binding may result in misdiagnosis or missed diagnosis. INPs can be designed with higher stability and specificity for enhanced imaging accuracy and reliability. Some nanomaterials, such as silicon nanomaterials, carbon nanomaterials (nanotubes, graphene, etc.), metal sulfur QDs [usually bound to zinc (II), cadmium (II), Selenide, sulfide), upconversion nanoparticles (UCNPs) (erbium (III), thulium (III), ytterbium (III), etc.] [33] as fluorescent contrast agents can achieve early detection of breast cancer via active or passive targeting. In addition, by selecting different surface modifiers or functional molecular links, QDs can bind specifically to the biomarkers of breast cancer cells, thus achieving a higher accuracy of early diagnosis [83–85].

Dai et al. compared the fluorescence contrast effects of erbium-based rare-earth nanoparticles ErNPs-TRC105 (an antibody to CD105 on tumor vasculatures) with IRDye800-TRC105, which is used in human clinical trials for tumor or sentinel lymph nodes localization [35]. The result indicated that at 24 h post-injection, mice injected with ErNPs-TRC105 demonstrated a high tumor NIR-IIb emission signal with a low background signal over the body (5 ms of exposure time), compared to the high background body signal for mice injected with IRDye800-TRC105.

Zhan et al. developed a photo-triggered cycloaddition reaction via bMSN@T2-RGD-Acrk, a non-toxic, biocompatible fluorescent silica nanoprobe, demonstrating a strong affinity for 4T1 breast cancer cells with significant accumulation of cytoplasm [86]. Notably, in vivo FLI performed 4 h after the intravenous injection of the nanoprobes revealed that bMSN@T2-RGD-Acrk exhibited higher fluorescence intensity in transplanted orthotopic 4T1 tumors. STBRs (signal-to-background ratios) of bMSN@T2-RGD-Acrk were nearly 2.4-fold greater than bMSN@T2-AM, suggesting that bMSN@T2-RGD-Acrk, as a targeted fluorescent nanoprobe, has significant binding and imaging capabilities for breast cancer cells, thus holding substantial value for the diagnosis and monitoring of breast cancer (Fig. 4D).

Chen et al. synthesized CQD-KD1, a CQDs conjugated with a recombinant st14 inhibitor (KD1) to target MCF-7 breast cancer cells, which are known for overexpressing st14 on the cell surface [66]. CQD-KD1 exhibits robust fluorescent imaging features, showing broad emission spectra with peak emissions around 545 nm when excited at 450 nm. Furthermore, CQD-KD1 demonstrates exceptional photostability, maintaining fluorescence intensity even after 10 h of continuous irradiation of household light (12.5 mW/cm^2^), significantly outperforming traditional small molecule probes like FITC. They determined the cellular imaging of CQD-KD1 at ex488 and ex546. After pre-incubating with 6.5 mg/mL CQD-KD1, MCF-7 cells exhibited strong fluorescence with the above two channels. The fluorescence was closely aligned with cell membranes, indicating the precise localization of the CQD-KD1. These results indicated that INPs could present a valuable tool in the medical imaging of breast cancer.

The integration of INPs in FLI enables the utilization of highly selective and stable fluorescent probes for specific binding to breast cancer cell biomarkers via active or passive targeting. This approach offers real-time detection and high sensitivity in breast cancer diagnosis, thereby enhancing the accuracy of early detection. However, FLI is influenced by factors such as the selectivity and stability of fluorescent probes and limitations in fluorescent signal penetration depth, which may restrict its ability to detect deep tissues or small micrometastasis. Consequently, researchers focus on improving signal penetration and optimizing probe design via nanotechnology to enhance stability and targeting capabilities. Simultaneously, the multimodal integration of FLI with other imaging technologies like MRI or CT and the development of new NIR FI technology can significantly improve imaging depth and resolution, providing a more comprehensive understanding of disease information during early breast cancer diagnosis.

### Surface-enhanced Raman scattering

SERS is performed as a transformative technique that, when integrated with INPs, offers remarkable capabilities in the early diagnosis, precise subtyping, and even the treatment monitoring of breast cancer. The unique fingerprint provided by SERS, characterized by very narrow peaks for individual molecules, allows for high accuracy and the simultaneous detection of multiple analytes, thereby significantly advancing the field of breast cancer diagnostics [87, 88].

Bardhan et al. demonstrated an accurate SERS-based detection of TNBC biomarkers both in vivo and in vitro by conjugating Raman tags and monoclonal antibodies specific to PD-L1 and EGFR onto the surface of gold nanostars (AuNS) [62]. Multiplexed SERS longitudinal study of the functionalized AuNS was performed after administering retro-orbital injections to nude mice bearing MDA-MB-231 TNBC xenografts. They pre-blocked both PD-L1 and EGFR as negative controls. Raman signals of pre-blocked groups decreased by about 30% compared with the unblocked group, indicating that the nanomaterial is sensitive and specific to distinguish breast cancer with different expression levels of PDL1 and EGFR. This labeled-AuNS could also accurately detect the expression status of different biomarker signals in the same tissue slice in vitro. Furthermore, the electron microscopy results demonstrated that the nanomaterial was endocytosed by tumor, liver Kupffer cells, and spleen macrophages, indicating that it could be used for SERS imaging and removed from the body (Fig. 4E).

Jia et al. constructed a SERS chip based on Ag_2_O–Ag–Psi to rapidly detect breast cancer [89]. In this platform, the Ag_2_O–Ag nano core shell, with a diameter of 40–60 nm, is embedded in a porous silicon substrate. Compared with the conventional Raman spectrum, the SERS spectrum of breast cancer patient’s serum samples greatly improved the intensity of multiple leading spectral bands representing diverse biochemical components. By calculating 10 data points at 1157 cm^–1^, 1518 cm^–1^, 1153 cm^–1^, and 1516 cm^–1^, the relative standard deviations of the intensity of Raman shifts were 4.6%, 5.1%, 2.5%, and 3.1%, respectively, which indicate that SERS signals exhibit excellent consistency for accurate breast cancer diagnosis.

The expression of several serum exosome-derived miRNAs is correlated with different breast cancer subtypes and could serve as potential biomarkers for breast cancer diagnosis. Sim et al. developed a SERS (based on AU) sensing platform for quantitatively determining exosomal miRNAs [90]. To evaluate the efficiency and accuracy of the developed SERS sensor, they conduct a recovery test by adding known concentrations of miR-21, miR-212, and miR-200c to human serum. Using this SERS-based sensor, they obtained high analytical recovery rates of these miRNAs as 95.28%, 101.68%, and 98.34%, respectively, with a relative standard deviation of 3.03%, 4.72%, 3.61%, respectively. Data measured by this sensor are highly consistent with the PCR results, suggesting the potential use of INPs-modified SERS sensor in the classification diagnosis of breast cancer.

Maiti et al. utilized AuNPs, which measured 40–45 nm in size, and served as the SERS substrate for the development of Raman-label surface-enhanced Raman scattering (RL-SERS)-nanotags [91]. SERS nanotags demonstrated significant sensitivity and specificity in detecting breast cancer biomarkers across different cell lines. SERS nanotags effectively identified respective biomarkers for the MCF-7 cell line (ER and PR positive) and the SK-BR-3 cell line (HER2 positive). In the triple-negative MDA-MB-231 cell line (ER−/PR−/HER2−), they showed negligible expression, confirming their specificity. In the tissue analysis, the sensitivity and specificity for single biomarker detection were 95% and 92%, respectively, 88% and 85% for duplex, and 75% and 67% for triplex analysis. The study also demonstrated the capability of SERS nanotags in the HER2 grading of breast cancer tissue samples, differentiating between 4+/2+/1+ HER2 expression levels with Raman intensity ratios of 3.67 ± 0.51, 2.17 ± 0.2, and 1.75 ± 0.15, respectively, correlating well with fluorescent in situ hybridization (FISH) analysis.

These results indicated that SERS shows excellent potential in the early diagnosis and precise typing of breast cancer. Combining with INPs, SERS not only plays a key role in the diagnosis of breast cancer but also shows significant value in efficacy diagnostics.

### Biomarker blood test

Blood biomarkers, such as carbohydrate antigens 15-3 (CA15-3), carcinoembryonic antigen (CEA), CA 27.29, HER2/neu, and CTCs, are primarily used to clinically assist in diagnosis and monitor the response to the treatment or recurrence of breast cancer [93–96]. However, standard laboratory medical testing techniques, including ELISA or other immunochemical methods, often fall short in early screening due to their limited sensitivity and specificity. INPs offer more accurate detection and quantification of breast cancer biomarkers at lower concentrations with enhancing fluorescence [97, 98]. In addition, the flexible customization of surface properties improves the selective adsorption with specific breast cancer antigens [91], thus facilitating an accurate early diagnosis and provision of precision medicine, significantly impacting treatment outcomes [99]. Table 2 summarizes the applications of nanomaterials for breast cancer blood tests and corresponding targets in past years.

**Table 2:** Summary of nanomaterials for breast cancer blood test and corresponding targets

Biomarker	Target base	Inorganic nanomaterial	Minimum detected concentration	Ref.
AFP	Anti-AFP antibody	GOQDs	0.01 U/mL	[105]
Beta‐1	DNA probes	AuNPs	1.2 nM	[106]
BRCA-1	DNA probes	SiO_2_@Ag/dsDNA/RhB	2.53 fM (S/N = 3)	[107]
CA 27‐29	Immunosensor	Au/MoS_2_/rGO nanocomposites	0.08 U/mL	[108]
CA125	Anti-CA125 antibody	Graphene	0.04 mU/mL	[109]
CA153	Anti-CA153 antibody	Ag NPs	0.01 U/mL	[92]
CA199	Anti-CA199 antibody	GOQDs	0.01 U/mL	[105]
CD63	Aptamer	MPA-CdS:Eu NCs	7.41 × 10^4^ particles/mL	[110]
CEA	Anti-CEA antibody	Ag NPs	1 pg/mL	[92]
Con A	Mannose	Man-BSA-Au NCs	0.62 nM (S/N = 3)	[111]
HER2	Aptamer	AuNPs	0.0904 fM	[112]
Let-7b	DNA probes	AgNPs@Si	~ 1 aM	[113]
miR‐199a‐5p	DNA probes	GO and GNR	4.5 fM	[114]
miR-1	DNA probes	AgNPs@Si	~ 1 aM	[113]
miR-10b	DNA probes	AgNPs@Si	~ 1 aM	[113]
miR-1246	DNA probes	AuAgNP	1.0 nM	[115]
miR-125b	DNA probes	AgNPs@Si	~ 1 aM	[113]
miR-126	DNA probes	AgNPs@Si	~ 1 aM	[113]
miR-133a	DNA probes	AgNPs@Si	~ 1 aM	[113]
miR-143	DNA probes	AgNPs@Si	~ 1 aM	[113]
miR-155	DNA probes	AgNPs	20 zmol	[116]
miR-21	DNA probes	AuNPs	5 fM	[117]
miR-221	DNA probes	AuAgNP	1.0 nM	[115]
miR-34a	DNA probes	AuNS	10 ng/10 μL	[118]
miR-375	Single-stranded DNAs	AuNP	0.36 fM	[119]
miR-K12-5-5p	DNA probes	GaN nanostructures with Au/Ag	8.84 × 10^−10^ M	[120]
miRNA-122	DNA probes	AuHFGNs/PnBA-Mxene	0.0035 aM	[121]
miRNA-141	DNA probes	SA@GNPs and Au@MNPs	1.8 pM	[122]
miRNA-652	DNA probes	AuNP	2.91 fM	[123]
MUC‐1	Aptamer	AuNPs and GO	0.031 fM	[124]
tPA	tPA monoclonal antibody	SWCNTs	0.026 ng/mL	[103]

AFP: alpha-fetoprotein; Beta‐1: beta protein 1; BRCA-1: breast cancer susceptibility gene 1; CA 27‐29: carbohydrate antigen 27–29; CA125: carbohydrate antigen 125; CA153: carbohydrate antigen 153; CA199: carbohydrate antigen 19-9; CD63: cluster of differentiation 63; Con A: concanavalin A; HER2: human epidermal growth factor receptor 2; Let-7b: lethal-7b; MUC‐1: mucin 1; GOQDs: graphene oxide quantum dots; RhB; rhodamine B; rGO: reduced graphene oxide; MPA-CdS:Eu NCs: mercaptopropionic acid (MPA)-modified Eu^3+^-doped CdS nanocrystals, Eu: europium; Man-BSA-Au NCs: mannose functionalized bovine serum albumin (BSA) encapsulated Au NCs; GaN: gallium nitride; AuHFGNs/PnBA-Mxene: hierarchical flower-like gold, poly (n-butyl acrylate), and MXene; SA@GNPs: silica-coated, analyte-tagged gold nanoparticles

Cui et al. introduced a microfluidic biosensor that combined CA153, CA125, and CEA antibodies with DTNB, 4-mercaptobenzoic acid (4MBA), and 2-naphthalenthiol (2NAT)-labeled Ag nanomaterials to detect corresponding antigens for breast cancer diagnosis [92]. This microfluidic biosensor could perform a reliable quantitative analysis of blood biomarkers in actual samples, and the detection results were consistent with those of commercial ELISA kits. The limits of detection (LOD) for CA153, CA125, and CEA in serum with the ELISA technique were found to be 0.028 U/mL, 7 U/mL, and 0.02 ng/mL in PBS, respectively [100–102]. Although microfluidic biosensor based on INP achieved a test sensitivity of 0.01 U/mL, 0.01 U/mL, and 1 pg/mL, it strongly highlights the application value of this microfluidic chip in an early diagnosis of breast cancer (Fig. 4F).

Madrakian et al. developed a biosensor utilizing single-wall carbon nanotubes (SWCNTs) covalently linked to the monoclonal antibodies for tissue plasminogen activator (tPA) [103]. This biosensor demonstrated a linear response range from 0.1 to 1.0 ng/mL with a remarkably low detection limit of 0.026 ng/mL, crucial for early breast cancer detection. Compared to traditional methods like HPLC and ELISA, this biosensor accurately detected low-concentration biomarkers in serum and is suitable for early auxiliary breast cancer diagnosis.

Zheng et al. developed a homogenous Magneto-Fluorescent Exosome (hMFEX) nanosensor for the rapid and on-site analysis of tumor-derived exosomes [104]. This nanosensor was used to detect exosomes in a dynamic range spanning five orders of magnitude with a LOD of 6.56 × 10^4^ particles/µL. The hMFEX nanosensor could analyze tumor-derived exosomes in 80 μL of centrifugal plasma from breast cancer patients, demonstrating excellent clinical diagnostic efficacy (AUC = 0.950, sensitivity = 86.11%, specificity = 90%). This study highlights the value of the nanosensor in diagnosing breast cancer, particularly in point-of-care approach.

Integrating INPs in blood tests for breast cancer diagnostics improves the accuracy and speed of diagnostic processes. It also opens new avenues for non-invasive, real-time monitoring of disease progression and treatment response. This innovative approach holds great promise for the future of cancer diagnostics, potentially transforming patient care and improving survival rates.

Currently, the applications of INPs in breast cancer diagnosis show excellent promise for overcoming these limitations compared to conventional diagnostic methods. Using INPs as contrast agents can enhance image clarity and specificity in MRI/CT/PET scans while improving imaging contrast and fluorescence signal stability in PAI/FLI scans. Furthermore, INPs can improve the detection sensitivity and specificity when applied to SERS technology or blood biomarker testing in clinical laboratories. The future development and optimization of nanotechnology are expected to enhance the critical role of these materials in improving breast cancer diagnosis accuracy while reducing costs and invasiveness and enhancing patient comfort.

## INPs for the treatment of LABC

Practical guidelines for treating LABC, especially TNBC, are still lacking [125, 126]. Drug resistance to chemotherapy, the inability to reuse radiotherapy after a specific dose, and tumor recurrence after surgery, leading to difficulty in secondary resection, are some major obstacles. Thus, novel methods must be investigated urgently.

### Radiotherapy

**Fig. 5:**
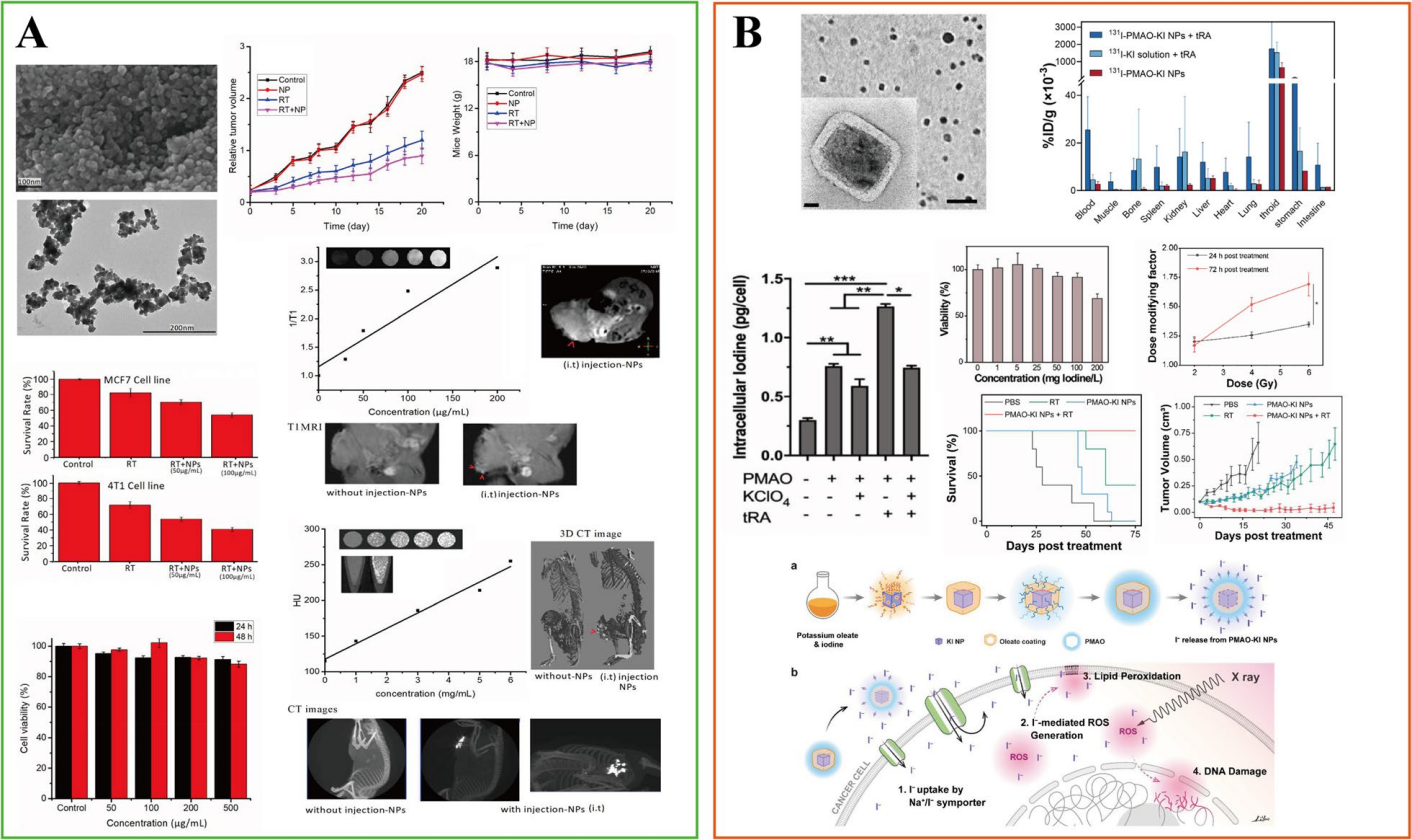
INPs used in breast cancer radiotherapy. **A** BiGdO_3_-PEG NPs; **B** PMAO-KI NPs. **A** Is adapted with permission from [135], copyright 2019 Physics in Medicine & Biology. **B** Is adapted with permission from [144], copyright 2021 ACS Nano

Radiotherapy, as an effective treatment for the local control of LABC, also has potential or acute side effects [127]. Developing new biosafe radiosensitizers is an effective solution. INPs-based radiosensitizers can enhance the susceptibility of tumor cells to ionizing radiation, resulting in DNA damage and the inhibition of DNA repair while increasing the oxidative stress level to induce autophagy and apoptosis. In addition, the occurrence of other biological effects, such as cell cycle inhibition and endoplasmic reticulum stress, ultimately led to cell death and improved the efficacy of radiotherapy [128–130]. In recent years, the application of INPs in radiotherapy focuses on high Z materials, primarily gold, silver, platinum, Gd, and so on, which can amplify radiation dose deposition due to their high atomic numbers and strong photoelectric absorption coefficient [129, 131–133]. At the same time, high Z materials, especially metals, tend to be chemically inert, which could reduce damage to healthy cells. Based on the favorable biocompatibility and excellent radiosensitization potential of these metal nanomaterials, Yook et al. developed Au NPs linked to *β*-particle emitter ^177^Lu and panitumumab to create ^177^Lu-T-AuNP as novel neoadjuvant brachytherapy for LABC [134]. In long-term monitoring, ^177^Lu-T-AuNP-based radiotherapy arrested tumor growth, which stopped after 90 days of treatment with no normal tissue toxicity, whereas mice’s survival time was extended to 120 days. Their work proposed a new approach for applications in LABC. In another study, Rajaee et al. reported the radiosensitizing ability of PEG-modified bismuth gadolinium oxide (BiGdO_3_-PEG NPs) in MCF-7 and 4T1 breast cancer cells (Fig. 5A) [135]. BiGdO_3_-PEG NPs could enhance the radiosensitivity of MCF-7 and 4T1 cell lines, with a radiation sensitizer enhancement ratio (SER) of 1.75 and 1.6, respectively. BiGdO_3_-PEG NPs can effectively inhibit the growth of tumor cells under low-dose irradiation. Moreover, more high Z metals, including nanoparticles with gold silicon shells as the core (AuN@SiO_2_ and AuS@SiO_2_) [136], nanoparticles coated with ultrasmall gold nanocrystals (Au@Cu-Sb-S) [137], cerium oxide nanoparticles coated with the anti-cancer drug neotenic acid (NGA-CNPs) [138], highly biocompatible poly (vinylpyrrolidone)-coated Ta nanoparticles (Ta@PVP NPs), have been identified as candidates for improved LABC radiation therapy [139].

As a recognized radiosensitizer, iodine has been used in clinics since the last century [140–142]. However, its short half-life and low rate of tumor retention have limited its application [143]. Cline et al. designed the poly maleic anhydride-alt-1-octadecene (PMAO)-coated KI nanoparticles (PMAO-KI NPs). For the first time, they evaluated the potential of KI NPs as a radiosensitizer to enhance radiotherapy in MCF-7 breast cancer cells (Fig. 5B) [144]. Their work employed the Na^+^/I^−^ symporter (NIS) for the iodine uptake delivery and radiosensitization, whereas NIS is expressed in most breast cancer cells. The polymer coating extends the half-lives of the KI NPs. The results demonstrated that PMAO encapsulation doubled the intracellular iodine content compared to the control group. Moreover, after using trans-retinoic acid (tRA) to promote the expression of NIS, the intracellular iodine level was further enhanced to achieve about 1.25 pg/cell, which can exert a radiosensitizing effect. In the presence of tRA, the cell survival rate of PMAO-KI NPs after 5 Gy irradiation was further reduced by 34.22% compared to that without tRA. Next, radiation therapy was performed following the injection of PMAO-KI NPs intrathecally in an MCF-7 tumor-bearing mice model. The results exhibited significant tumor regression. Patients with LABC often receive multiple radiotherapy before surgery. The half-life of PMAO-KI NPs and intracellular iodine content can be increased to achieve sustained iodide release and radiosensitization by modifying the appropriate coating thickness; thus, one injection that can benefit multiple radiotherapy sessions will be possible.

Radiotherapy is still a common clinical strategy for the management of LABC. Developing INPs with high biocompatibility and excellent sensitization effects is urgently needed to ameliorate the existing radiotherapy situation.

### Phototherapy

Phototherapy is selective, adjustable, and non-invasive. Its application scope is limited because the penetration capacity and depth of the laser usually are less than 5 cm [145]. However, as an accessory organ of the human body, the lesion mammary gland is relatively shallow compared with other organs, making phototherapy undoubtedly suitable for treating LABC [146, 147]. Meanwhile, compared with radiotherapy, phototherapy uses a low-energy laser and minimizes skin toxicity, which is a good option for those who can no longer receive radiation [148]. At present, phototherapy primarily focuses on PTT and PDT. PTT uses the power of photothermal agents (PTAs) to absorb light and convert energy into heat [149]. The temperature range of photo-induced hyperthermia is about 40–48 °C, which can achieve the purpose of tumor ablation while minimizing damage to adjacent healthy cells [147, 150]. However, PDT uses PSs to interact with active biomolecules under light excitation to produce reactive oxygen species (ROS) to kill tumor cells by inducing apoptosis, necrosis, and autophagy [151].

Besides, researchers demonstrated that INPs have favorable physical and chemical properties, stability, biocompatibility, upconversion characteristics, etc., which are competent in the role of PSs and PTAs with perfect application potential [152, 153]. When we choose the light source, NIR light is undoubtedly a suitable and potential one. Biological transparency windows NIR-I (750–1000 nm) and NIR-II window (1000–1700 nm) have high tissue penetration and retention with low side effects [154, 155]. The combination of INPs and NIR light has shown promising results in treating LABC. This includes but is not limited to the following materials.

#### Photothermal therapy

**Fig. 6:**
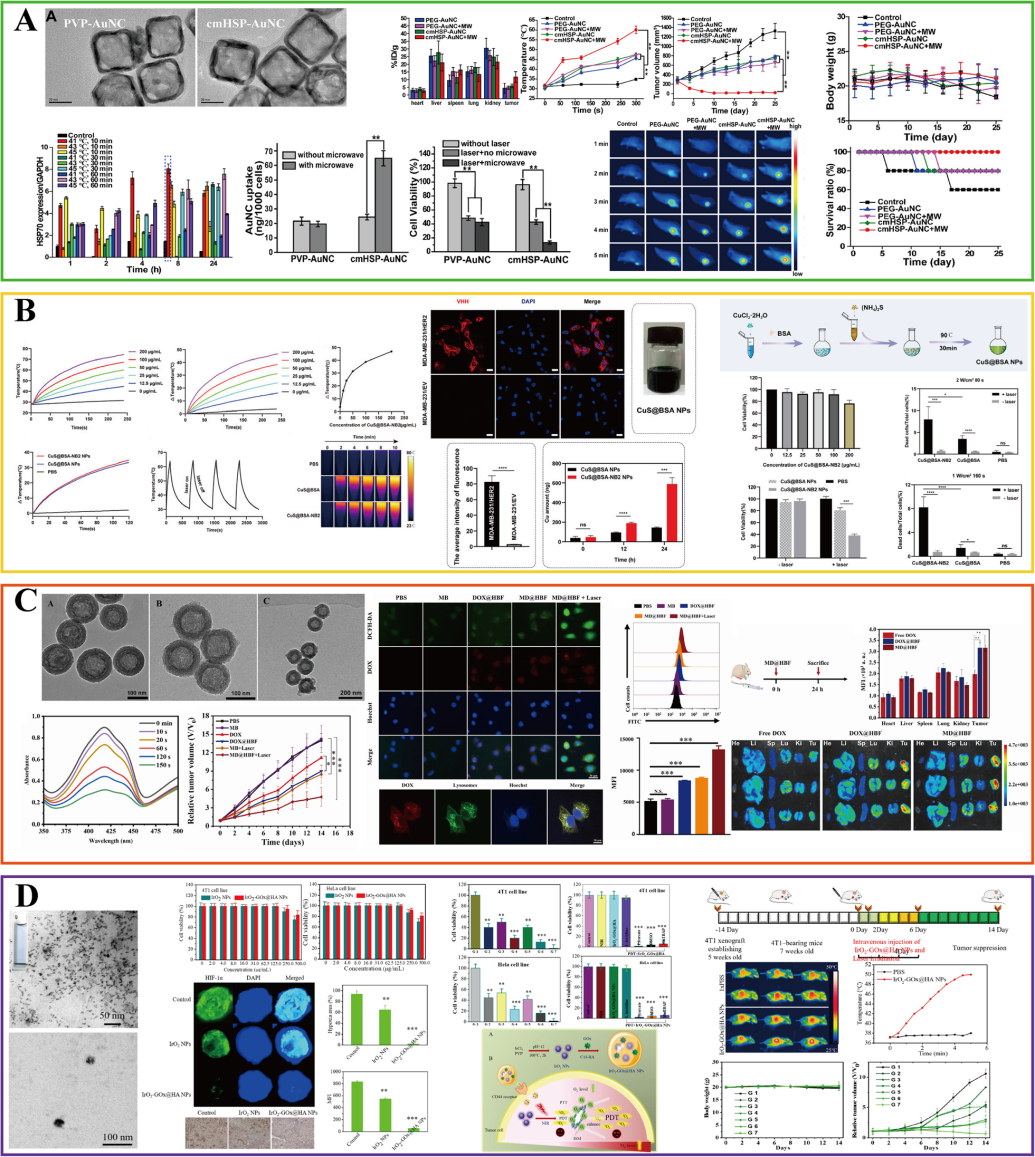
INPs used in breast cancer phototherapy. **A**, **B** The application in photothermal therapy: **A** cmHSP-AuNC; **B** CuS@BSA-NB2. **C**, **D** The application in photodynamic therapy: **C** MD@HBF; **D** IrO_2_-GOx@HA NPs. **A** Is adapted with permission from [161], copyright 2021 International Journal of Pharmaceutics. **B** Is adapted with permission from [178], copyright 2022 Frontiers in Pharmacology. Panel **C** Is adapted with permission from [200], copyright 2023 Colloids and Surfaces B: Biointerfaces. **D** Is adapted with permission from [205], copyright 2022 Journal of Colloid and Interface Science

PTT relies on efficient photothermal conversion agents. Among the many photothermal conversion agents, noble metal materials, including Au, Ag, Pt, and Pd, are the most extensively investigated due to their antioxidant properties [156]. Gold nanomaterials have been studied the most due to the advances in synthesis, suitable absorption, and strong stability under biologically relevant conditions [157, 158]. Meanwhile, gold-based nanomaterials had already been employed in the research on PTT for breast cancer as early as 2003 [159]. Hirsch et al. first designed the gold–silica nanoshells for the PTT in SK-BR-3 cells. After the irradiation by NIR (820 nm, 4 W/cm^2^) for 4–6 min, the temperature of the tumor tissue increased by 37.4 ± 6.6 °C, which could cause irreversible damage to tumor cells. LABC is classified as TNBC in approximately one-third of cases [160]. To develop a novel photothermal conversion agent for the treatment of TNBC, Cheng et al. designed a novel microwave-triggered heat shock protein (HSP)-targeted gold nanosystem (cmHSP-AuNC) to improve the accumulation of gold nanomaterials by specifically targeting 4T1 cells (Fig. 6A) [161]. Microwave irradiation triggered the overexpression of HSP in 4T1 cells, at which time anti-HSP monoclonal antibodies in the nanosystem can improve the accumulation of nanomaterials in 4T1 cells [162, 163]. After treatment by 808-nm NIR (1.0 W/cm^2^), the tumor inhibition rate of microwave-triggered cmHSP-AuNC was 98.48%, which was significantly higher than that in the non-microwave stimulated group (44.20%). As PTT research advances, the current trend is to develop INPs with lower laser energy consumption while maintaining higher photothermal conversion efficiency. Li et al. prepared core–hell nanostars (AuNS@CP NPs) with a AuNS core and a metallic drug coordination polymer (CP) shell [164]. AuNS@CP NPs have a temperature increase of about 35 °C under 808-nm irradiation (0.5 W/cm^2^, 3 min), which could produce significant photothermal ablation ability, killing 96% of 4T1 cells.

In addition, many other materials, such as sulfide, graphene, and transition metals, including QDs, metal oxide NPs, exhibited high photothermal conversion efficiency and have the potential in LABC treatment [165–168]. For instance, MnFe_2_O_4_ [169], AS-BSA-MnO_2_ [170], ultrafine graphene oxide (UGO) [171], PDA-DTC/Cu-MnO_2_ [172], CuS, Cu_2_−_*x*_S, and Cu_2_−_*x*_Se [173–176] are up-and-coming candidates for the treatment of breast cancer. Surface modification strategy is an important approach to improve the biocompatibility of NPs [177]. For example, Ying et al. developed a novel biocompatible nanoparticle CuS@BSA-NB2 for HER2-positive breast cancer treatment (Fig. 6B) [178]. CuS is modified by BSA to decrease cytotoxicity and conjugated with the HER2 nanobody (NB2) to create the CuS@BSA-NB2 nano-complex. In this work, CuS@BSA-NB2 has shown the ability to specifically target MDA-MB-231/HER2 cells. As an excellent PTA, CuS@BSA-NB2 could rapidly reach 59 °C under 808-nm (1 W/cm^2^, 4 min), effectively killing more than 60% of MDA-MB-231/HER2 cells. Summarily, their work presented fresh insights on the treatment of HER2-positive breast cancer. PTT showed application potential in the treatment of LABC. Representative examples of INPs applied to the PTT for breast cancer treatment in the past decade are briefly summarized in Table 3, providing insights into INPs preparation strategies to overcome different breast cancer subtypes.

**Table 3:** Representative application of INPs-based PTT in breast cancer with various subtypes

Type of INPs	Size (nm)	Laser wavelength (nm)	Breast cancer cell type (subtype)	Cell safety concentration (incubation time)	Animal tumor model	ΔT	Laser settings	Ref.
GNRs-MPH^−ALA/DOX^-PEG)	42.9	808	MCF-7 (luminal A)	20 μg Au/mL (24 h)	Subcutaneous MCF-7 mice model	30 °C	2 W/cm^2^ (20 μg Au/mL, 10 min)	[179]
PEG@Pt/Dox	94	808	MCF-7 (luminal A)	80 μg/mL (48 h)	None	> 30 °C	1 W/cm^2^ (40 μg/mL, 5 min)	[180]
GNR-BBN-PEG	L: ~ 50	830	T47D (luminal A)	100 μg GNRs/mL (24 h)	Subcutaneous mice model	None	None	[181]
^177^Lu-DenAuNP-folate-bombesin	2.5 ± 0.4 (Au NPs)	532	T47D (luminal A)	None	None	9.8 °C	1.1926 W/cm^2^ (none, 6 min)	[182]
FeOxH-rGO	~ 300 (rGO)	808	T47D (luminal A), 4T1 (TNBC)	300 µg/mL	Subcutaneous 4T1 mice model	44 °C	1.82 W/cm^2^ (300 μg/mL, 5 min)	[183]
PLL-Au–Fe_3_O_4_ NPs	55 ± 8.6 (Au–Fe_3_O_4_ NPs)	808	BT-474 (luminal B/HER2^+^), MDA-MB-231 (TNBC)	100 μg/mL (2 h)	None	> 25 °C	1 W/cm^2^ (800 μg/mL, 10 min)	[184]
DIR-SPIO-PLGA/PFP NPs	298	808	SK-BR-3 (HER2^+^), MDA-MB-231 (TNBC)	2 mg/mL (24 h)	Subcutaneous SK-BR-3 mice model	> 54.9 °C	2 W/cm^2^ (11.25 mg/mL, 10 min)	[185]
UCNP-PMAO-PVCL	30 (UCNPs)	980	SK-BR-3 (HER2^+^), MDA-MB-231 (TNBC)	2 μg/mL (72 h)	Subcutaneous SK-BR-3 mice model	~ 3.5 °C	0.5 W/cm^2^ (0.4 mg/mL, 1 min)	[186]
Pd Ncap-Her	~ 113 (Au bead@Ag nanorods)	1064	SK-BR-3 (HER2^+^)	100 µg/mL (24 h)	None	~ 38 °C	3 W/cm^2^ (50 µg/mL, 6 min)	[187]
DOX-PEG-NCs	199.6 (PEG-NCs)	808	MDA-MB-453 (HER2^+^)	0.1 mg/mL (72 h)	None	28 °C	2.027 W/cm^2^ (400 ppm, 10 min)	[188]
AuNUs	155.0 ± 1.0	808	MCF-7, (luminal A) MDA-MB-231, MDA-MB-468 (TNBC), and MDA-MB-453 (HER2^+^)	100 µg Au/mL (48 h)	None	21 °C	3.5 W/cm^2^ (50 µg/mL, 3 min)	[189]
TAgNPs	93.33 ± 1.63	970	MDA-MB-231 (TNBC)	50 µg/mL (72 h)	None	> 30 °C	3 W (12.5 µg/mL, 1 min)	[190]
LCP-CD/ICG-BsAb NPs	62 ± 8	808	MDA-MB-468 (TNBC)	None	Subcutaneous MDA-MB-468 mice model	~ 20 °C	0.5 W/cm^2^ (20 µM ICG, 4 min)	[191]
HCIONPs	24.4	885	SUM-159 (TNBC)	None	Subcutaneous SUM-159 mice model	33 °C	2.5 W/cm^2^ (0.5 mg Fe/mL, 10 min)	[192]
MnPB-MnO_x_ NPs	168.5 ± 3.1	808	4T1 (TNBC)	6.2 ppm (24 h)	Subcutaneous 4T1 mice model	~ 40 °C	1.5 W/cm^2^ (100 ppm, 10 min)	[193]
IRFes	334	808	4T1 (TNBC)	0.8 mg/mL (6 h)	Subcutaneous 4T1 mice model	30 °C	1 W/cm^2^ (2 mg/mL, 3 min)	[194]

L represents for length; none represents for that not specifically mentioned by the author in the paper; THE ‘laser settings’ on the right correspond to the ‘ΔT’ data on the left, representing the concentration of the selected INPs, laser power, and duration time in the photothermal performance test

MPH: mercaptopropionylhydrazide; ALA: 5-aminolevulinic acid; DOX: doxorubicin; BBN: bombesin; ^177^Lu-DenAuNP-folate-bombesin: ^177^Lu-DOTA-dendrimer-AuNP-folate-bombesin; FeOxH-rGO: iron hydroxide/oxide immobilised on reduced graphene oxide nanocomposites; PLL: poly-l-lysine; DIR: 1,1′-dioctadecyl-3,3,3′,3′-tetramethylindotricarbocyanine iodide; SPIO: superparamagnetic iron oxide; PLGA: poly lactic-co-glycolic acid; PFP: perfluoropentane; PVCL: poly-*N*-vinylcaprolactam; Pd Ncap-Her: palladium nanocapsule functionalized with Herceptin; AuNUs: L-dopa functionalized nanourchin-like AuNPs; TAgNPs: triangular silver nanoparticles; LCP-CD/ICG-BsAb NPs: the indocyanine green (ICG) and/or cell death (CD) siRNA-loaded lipid-coated calcium phosphate nanoparticles, functionalized with bispecific antibody (BsAb); HCIONPs: highly crystallized iron oxide nanoparticles; MnPB-MnO_x_ NPs: Mn-enriched photonic nanomedicine; IRFes: magnetically targeted nanoparticles

#### Photodynamic therapy

Because of its minimally invasive and selective characteristics, the applications of PDT in breast research have made significant progress. Traditional PSs like hematoporphyrin derivative and photofrin have more or less defects, such as low chemical purity, cutaneous phototoxicity, and long half-life [195, 196]. The accumulation of hydrophobic PSs represented by zinc phthalocyanine in an aqueous solution also affected the therapeutic objectives [197]. The construction of PS based on a nano platform can improve these problems. Due to their high stability, adjustable size, optical properties, and accessible surface functionalization, inorganic nanomaterials can be used as carriers to deliver PSs to achieve therapeutic effects [198]. For example, hypericin, an example of such PSs limited by its hydrophobicity, can enhance the hypericin uptake by MC-7 breast cancer cells via coupling with gold nanoparticles, thus improving the curative effect of PDT [199]. Moreover, the selectivity of breast cancer sites can be further enhanced by surface functionalization modification, like adding target groups or ligands. Zhang et al. designed a hollow mesoporous silica nanoparticle (HMSNs) coated with folic acid-modified BSA (BSA-FA) to form MD@HBF against folate receptor-expressing 4T1 cells [200]. HMSNs are used to deliver the PS and methylene blue to perform the PDT effect, and the BSA-FA structure increases the targeting ability of HMSNs (Fig. 6C) [201, 202]. This design helps to improve the tumor target ability and overcome the defects of traditional PSs.

Not only that, further research revealed that INPs themselves can also play a more critical role than PSs. At the same time, the photothermal conversion ability of inorganic nanomaterials can further enhance the therapeutic effect. It was first reported by Raviraj et al. that precious metal NPs can be sensitized directly to produce ^1^O_2_ without the use of organic PSs [203]. IrO_2_ NPs have the function of catalase, which can decompose endogenous H_2_O_2_ in the tumor microenvironment to generate ^1^O_2_ to relieve hypoxia, thus amplifying the therapeutic effect of PDT [204]. Yuan et al. combined hyaluronic acid (HA) and glucose oxidase (GO_X_) with iridium oxide nanoparticles to create an in situ amplifier: IrO_2_–GOx@HA NPs (Fig. 6D) [205]. In their study, elevated tumor glucose levels were enzymatically converted to H_2_O_2_ by GOx. Subsequently, IrO_2_ NPs facilitated the conversion of H_2_O_2_ into ^1^O_2_. The incorporation of HA enhanced the targeting selectivity toward 4T1 breast cancer cells, thereby augmenting the accumulation of ROS and ultimately boosting the PDT effect. Following the treatment with 808-nm NIR light, IrO_2_–GOx@HA NPs significantly killed more than 90% of 4T1 cells and inhibited tumor growth in vivo, showing a pronounced PDT effect. Meanwhile, IrO_2_ NPs with high photothermal conversion efficiency can further synergistically improve the curative effect [206].

Moreover, black phosphorus (B.P.), as a novel two-dimensional metal-free semiconductor, exhibits high biocompatibility, biodegradability, and excellent photocatalytic performance [207, 208]. Wang et al. designed black phosphorus nanosheets (B.P. nanosheets) for the PDT in breast cancer. They provided the initial empirical confirmation of its efficacy as a PS for generating ^1^O_2_, with a high quantum yield of about 0.91, which is higher than standard PS, rose bengal, suggesting its potential to be used in PDT [209, 210]. The apoptosis rate of MDA-MB-231 cells was 71.5% after light irradiation (660 nm, 1 W/cm^2^, 10 min). Finally, B.P. nanosheets effectively inhibited the growth of tumors in the MDA-MB-231 breast tumor-bearing mice model. Their results provide a new idea for investigating PSs of INPs.

In the early years, several sets of clinical trials evaluated the local control efficacy of PDT in breast cancer treatment [211, 212]. With the continued development of nanotechnology, it is not difficult to see a bright future for INPs-based PDT to overcome the challenges of LABC.

### Magnetic hyperthermia therapy

**Fig. 7:**
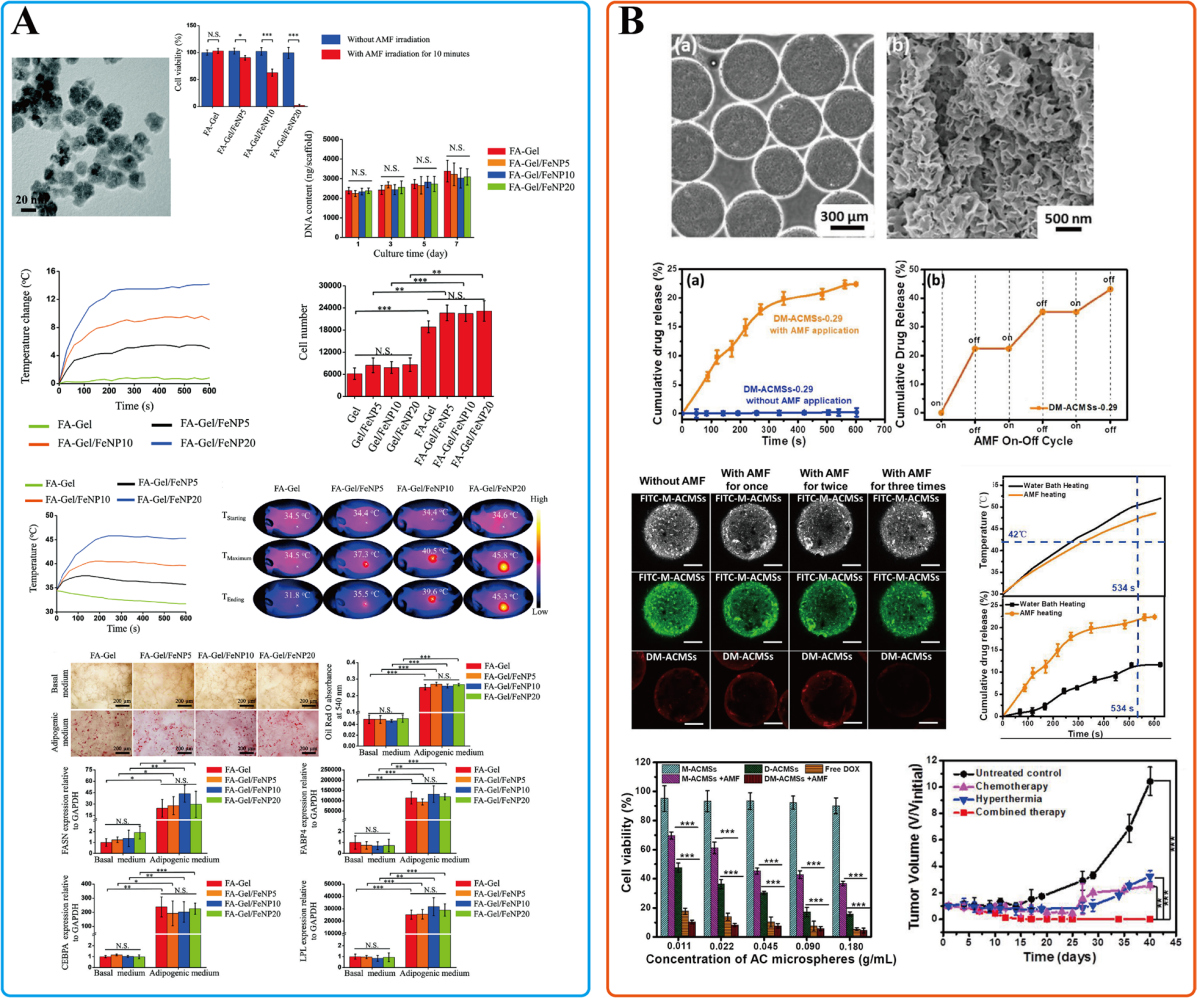
INPs used in breast cancer MHT. **A** FA-Gel/FeNP; **B** DM-ACMSs. **A** Is adapted with permission from [218], copyright 2023 Advanced Healthcare Materials. **B** Is adapted with permission from [221], copyright 2018 Journal of Materials Chemistry B

Hyperthermia combined with neoadjuvant therapy can enhance the therapeutic effect of patients with LABC [213]. NPs-based MHT offers a new method to overcome LABC: not just as an adjunctive treatment option, but as an independent therapy. Compared with laser, alternating magnetic field (AMF) has infinite tissue penetration ability, effectively stimulating NPs to achieve hyperthermia [214]. Among a wide range of different nanomaterials, iron oxide NPs are the most clinically oriented NPs and have been widely explored for MHT in breast cancer cells [215–217]. Sun et al. prepared AMF-responsive composite scaffolds (FA-Gel/FeNP) by using folic acid-modified gelatin and hybridized them with citrate-stable Fe_3_O_4_ NPs (Fe_3_O_4_-Citrate NPs) (Fig. 7A) [218]. This FA-functionalized composite scaffold has a large spherical hole and good interoperability, which can precisely capture MDA-MB-231-Luc breast cancer cells expressing FA receptors. Under the action of AMF (130 Gauss, 373.6 kHz), > 95% of tumor cells were killed. Moreover, FA-Gel/FeNP shows the supporting effect for stem cell differentiation to adipocytes, which has crucial implications for post-treatment or postoperative breast reconstruction.

In multi-group preclinical trials, the combination of hyperthermia and neoadjuvant chemotherapy has been essential in treating LABC [213, 219, 220]. Based on the good magnetic response of magnetic nanomaterials, controlled release of drugs can be achieved to play a synergistic therapeutic effect. Xue et al. developed AMF-responsive DOX-loaded magnetic microspheres (DM-ACMSs) for multimodality breast cancer treatment [221]. With the action of AMF, the temperature increased above 50 °C, up to 22.5% of DOX was released, and 95.5% of the MCF-7 breast cancer cells were killed. Furthermore, this innovative DM-ACMSs system showed on–off drug release capability by remotely controlling AMF (Fig. 7B). In addition, all tumors were eliminated in the combination treatment mode with no recurrence under the action of DM-ACMSs toward MCF-7 breast tumors. This AMF-responsive strategy could reduce tissue damage and cooperate with MHT to play an anti-tumor effect, which is a promising application in LABC preoperative treatment.

MHT has been extensively studied in different molecular types of breast cancer cell lines, and the AMF response-based hyperthermia and drug release mechanism are expected to improve the existing neoadjuvant therapy strategies, reduce tissue damage, and improve curative effect.

### Sonodynamic therapy

**Fig. 8:**
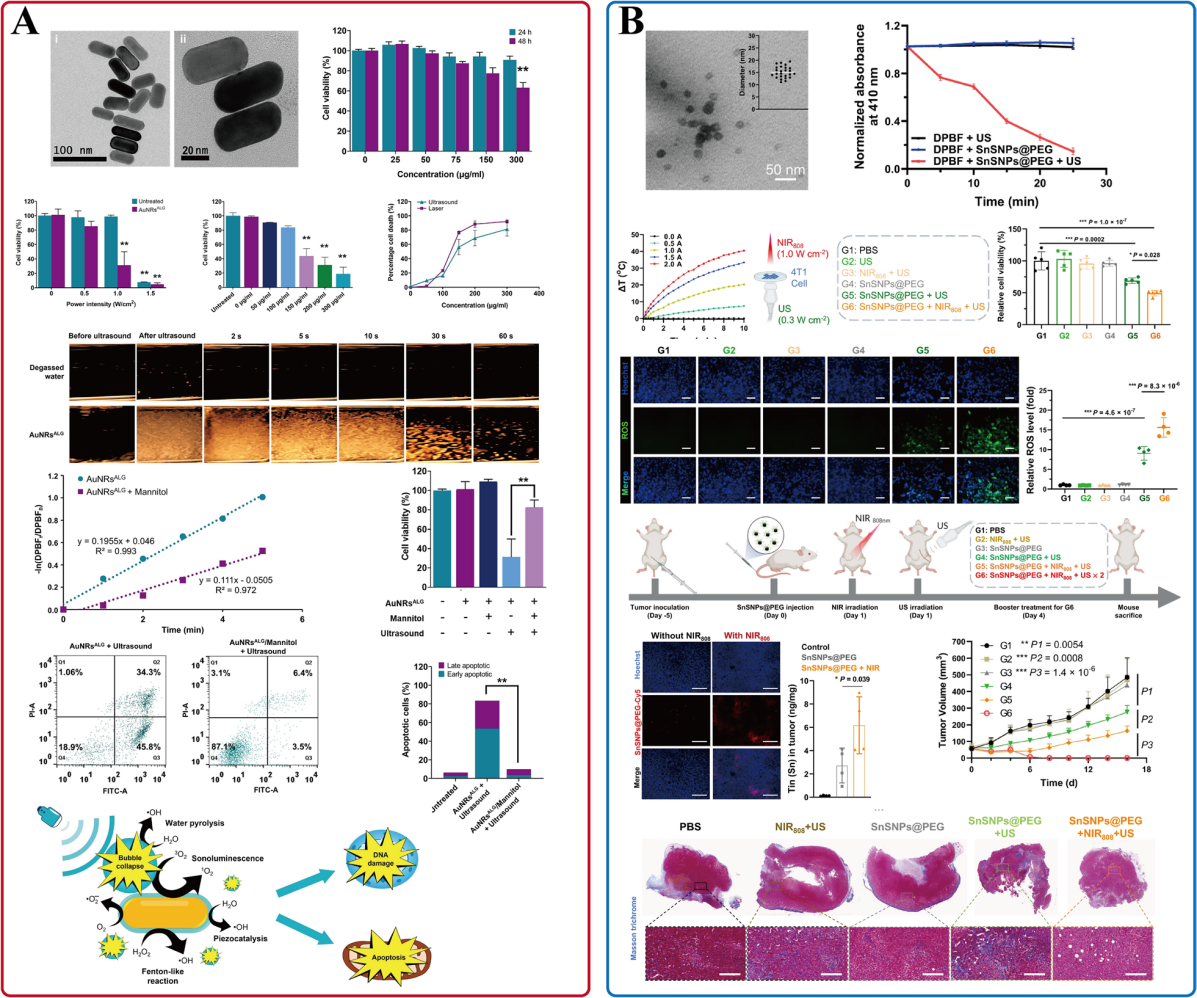
INPs used in breast cancer SDT. **A** AuNRs^ALG^; **B** SnSNPs@PEG. **A** Is adapted with permission from [228], copyright 2023 Ultrasonics Sonochemistry. **B** Is adapted with permission from [233], copyright 2023 Nature Communications

SDT, wherein sonosensitizers employed by US to catalyze the generation of ROS to kill cancer cells, has highly controllable, non-invasive, and deep tissue penetration ability (on the order of centimeters) [222–225]. This promising approach offers a fresh insight into LABC treatment. The execution of SDT hinges on the efficient separation of electron–hole (e^−^–h^+^) pairs within the US-activated sonosensitizers. The resulting e^−^–h^+^ pairs and the energy released from the activated sonosensitizers further react with surrounding O_2_ and H_2_O to generate cytotoxic ROS [226]. INPs characterized by stable chemical properties and prolonged circulation time in the blood can effectively reduce phototoxicity, demonstrating remarkable potential as sonosensitizers [227]. Loke et al. first reported the applicability of alginate-coated gold nanorods (AuNRs^ALG^) as promising sonosensitizers for SDT in breast cancer [228]. The results revealed that the AuNRs^ALG^ structure was stable under US irradiation (1.0 W/cm^2^, 5 min). Its ROS production rate constant was 1.96 × 10^–1^ min^−1^ (Fig. 8A), which is three- to eightfold higher than that of the previous studies, such as TiO_2_ nanospheres [229], Au–TiO_2_ nanosheets [230], or Au–TiO_2_ nanocomposites [231]. In vitro results demonstrated an 81% killing effect on MDA-MB-231 breast cancer cells. Furthermore, in vivo experiments are expected to verify its potential in LABC treatment.

Developing novel sonosensitizers with narrow bandgaps to effectively separate e^−^–h^+^ pairs is vital to enhance the generation of ROS in SDT [224, 232]. In another study, Li et al. designed a novel tin monosulfide NPs (SnS NPs) coated with PEG (SnSNPs@PEG) for the enhancement of SDT in 4T1 breast cancer cells [233]. In this work, SnSNPs@PEG with a narrow bandgap (1.18 eV) can produce ROS efficiently under the action of US (1 MHz, 1 W/cm^2^, 50% duty cycle). Simultaneously, SnSNPs@PEG exhibit the photothermal conversion capability, achieving a conversion efficiency of 25.2% under the irradiation of 808-nm NIR (2.0 W/cm^2^), which can denature tumor collagen, promoting the penetration of SnSNPs@PEG in the tumor, therapy improving the effect of SDT. The tumor was eradicated without recurrence in the 4T1 tumor-bearing mice (Fig. 8B). In conclusion, the SnSNPs@PEG-based therapeutic strategy could improve the accumulation of NPs in tumors and thus improve the therapeutic effect of SDT in breast cancer.

As a new, non-invasive method for treating deep tumors, SDT can increase patient compliance and improve the current treatment status of inoperable LABC through effective local control.

## INPs for the local theranostic application of LABC

With the innovation of modern nanotechnology, more and more inorganic nanomaterials tend to be multifunctional as efficient treatment and diagnosis platforms for LABC. Representative examples of INPs applied to the local diagnosis and treatment of breast cancer in the past years are summarized in Table 4, which can provide optimal preparation strategies for multifunctional nanoplatforms as required.

**Table 4:** Summary of breast cancer local theranostic applications based on inorganic nanoplatforms

Inorganic nanoplatform		Size (nm)	Cell type	Cell safety concentration (incubation time)	Animal tumor model	Application(s)	Ref.
Au	cGNPs	52.8 ± 22.0	MCF-7, HTB-123, and MDA-MB-231	3.5 OD (48 h)	Subcutaneous MDA-MB-231 mice model	RT	[250]
	Man@BAu NPs	597.5 ± 172.6	MDA-MB-231	0.1 mM (72 h)	None	PTT (808 nm)	[251]
	cmHSP-AuNC	61.2 ± 4.85	4T1	128 μg/mL (24 h)	Subcutaneous 4T1 mice model	PTT (808 nm)	[161]
	AuNRs^ALG^	69 ± 4	MDA-MB-231 and L929	300 μg/mL (24 h)	None	SDT	[228]
Ag	Ag_2_S-cRGD NPs	None	293 T, MCF-7, and 4T1	2.0 mM Ag^+^ (24 h)	Subcutaneous 4T1 mice model	PTT (808 nm)	[252]
	Ag_2_Te QDs	4.3	4T1	200 ppm (24 h)	Subcutaneous 4T1 mice model	CT/PTT (808 nm)	[39]
	Au–Ag/Ag–Au NPs	17 (Au–Ag) 15 (Ag–Au)	MDA-MB-231 and L929	50 μg/mL (48 h)	None	PTT (532/405 nm)	[253]
Cu	Au@Cu-Sb-S NPs	22.9 ± 1.8	4T1	100 ppm Sb (24 h)	Subcutaneous 4T1 mice model	CT/PAI/RT/PTT (808 nm)	[137]
Cd	pHSNNPs	100 ± 10	MCF-7 and L929	25 μg/mL (72 h)	None	FLI	[254]
	CdTe/CdS-5-FU, Bio-CdTe/CdS-TAM	2.9 (λ_em_ = 545 nm) 3.4 (λ_em_ = 600 nm)	MDA-MD-231	None	None	FLI	[255]
	GNR@CdSe/ZnS NPs	8 ± 1	MCF-7	None	None	FLI	[256]
Zn	NOTA-ZnO-PEG-TRC105	101.2 ± 10.7	4T1	None	Subcutaneous 4T1 mice model	FLI/PET	[257]
	PL-Au/PEG-ZnO NRs	L: 100 W: 18	MCF-7	20 μg/mL (24 h)	None	PDT (365 nm)	[258]
	Apt-NP-DOX	100	4T1, MCF-7, and CHO	None	Intravenous 4T1 mice model	FLI/MRI	[259]
	Z@C-D/P	210.3 ± 9.5	4T1 and NIH/3T3	1.25 μg/mL (36 h)	4T1 and NIH/3T3 mice model	PTT (808 nm)	[260]
Ti	N-GQDs/TiO_2_ NCs	49.2 ± 4.5	MDA-MB-231 and HS27	0.5 mg/mL (24 h)	None	PDT (700–900 nm)	[261]
	Avidin-TiO_2_ NPs	100 (TiO_2_ NPs)	MCF-7	None	None	SDT	[262]
Mo	FBPMP	133	MDA-MB-231 and L929	10 µg/mL (24 h)	None	PTT (808 nm)	[263]
	SP-MoS_2_	150.7–122.4	4T1 and L929	100 ppm (24 h)	None	PTT (808 nm)	[264]
UCNPs	NaYF4:Yb, Tm@TiO_2_/ZrO_2_–trastuzumab	6–8	SKBR-3 and MCF-7	400 μg/mL (24 h)	None	PDT (975 nm)	[265]
	FA-NPs-DOX	177	MCF-7 and MCF-7/ADR	100 μg/mL (48 h)	Subcutaneous MCF-7 and MCF-7/ADR mice model	PDT (980 nm)	[266]
Gd	GA-NPs	23.3 ± 1.2	4T1	4 μg/mL (24 h)	Subcutaneous 4T1 mice model	MRI/PDT/PTT (660 nm)	[267]
	BiGdO_3_-PEG NPs	11.3 ± 1.6	MCF-7 and 4T1	500 µg/mL (48 h)	4T1 mice model	CT/MRI/RT	[135]
Fe	Fe_3_O_4_-citrate NPs	94.2 ± 42.4	MDA-MB-231-Luc and HT1080	20 mg/cm^3^ (24 h)	Subcutaneous MDA-MB-231-Luc mice model	MHT	[218]
	CdSe/Fe_3_O_4_ NCs	200 ± 38	MCF-7, MDA-MB-231	0.1 mg/mL (24 h)	None	MHT/PTT (808 nm)	[268]
	G4@IONPs	10 ± 4	MC_4_L_2_	500 µg/mL (24 h)	Subcutaneous MC_4_L_2_ mice model	MHT	[269]
	Fe_3_O_4_-Au_shell_ NPs	25 ± 3.3	4T1	80 ppm (24 h)	Subcutaneous 4T1 mice model	MRI/PTT (808 nm)	[241]
Bi	Pt-Bi_2_S_3_ NPs	163	4T1, L929, and RAW 264.7 macrophages	400 µg/mL (24 h)	Subcutaneous 4T1 mice model	SDT	[270]
Sn	SnSNPs@PEG	32.6 ± 1.8	4T1, 4T1-luc, and RIL-175-luc	400 µg/mL (48 h)	Orthotopic 4T1 mice model	SDT/PTT (808 nm)	[233]
Si	M-MSN-HA/DI	355.3	MDA-MB-231, EMT-6, and NIH-3T3	80 µg/mL (48 h)	Subcutaneous MDA-MB-231 mice model	FLI/MRI/PAI/PTT (808 nm)	[235]
	Cet-SLN/ICG	100	MCF-7	0.5 μg/mL ICG (48 h)	MCF-7 mice model	PTT (808 nm)	[271]
	CSiFePNs	220	MDA-MB-231	0.1 mg/mL (48 h)	None	MHT	[272]
GPE	POx-GO	None	NHDF and MCF-7	100 µg/mL (48 h)	None	PTT (808 nm)	[273]
	IR/SBMA-BSA/GO	None	NHDF and MCF-7	75 μg/mL (48 h)	None	PTT (808 nm)	[274]
C	R-O_2_-FA-CHI-SWCNTs	None	MDA-MB-231 and ZR-75-1	100 μg/mL (48 h)	None	RT	[275]
	SWCNT-ANXA5	1.5 ± 0.5	EMT6	None	Intravenous EMT6 mice model	PTT (980 nm)	[276]
	MWNTs	L:1126 ± 389 (100 counts of MWNTs.)	MCF-7, MDA-231, and EMT6	100 μg/mL (24 h)	Orthotopically EMT6 mice model	PTT (808 nm)	[277]

L represents for length; W represents for width; ‘none’ represents for that not specifically mentioned by the author in the paper. RT represents for radiotherapy

cGNPs: CXCR4 monoclonal antibody-conjugated gold nanoparticles; Man@Bau NPs: mannoside-modified branched gold nanoparticles; cRGD: cyclic RGD peptide; pHSNNPs: pH-sensitive niosomal nanoparticles; 5-FU: 5-fluorouracil; TAM: tamoxifen; NOTA: 1,4,7-triazacyclononane-1,4,7-triacetic acid; TRC105: a monoclonal antibody that binds to CD105 (i.e. endoglin); PL: piperlongumine; Z@C-D/P: ZnO@CuS NPs, loaded with DOX and pirfenidone (PFD); N-GQDs/TiO_2_ NCs: N-doped graphene QDs/titanium dioxide nanocomposites; FBPMP: FA targeted dual-stimuli responsive MoS_2_ nanosheets (FA-BSA-PEI-LA (alpha-lipoic acid) -MoS_2_-LA-PEG); SP-MoS_2_: soybean phospholipid-encapsulated MoS_2_ nanosheets; GA-NPs: Gd_2_O_3_@albumin conjugating PS; G4: fourth generation of poly amidoamine (PAMAM); Cet-SLN: silica nanoparticles (SLN) conjugated with Cetuximab (Cet); CsiFePNs: system composed of MSNs containing Fe_3_O_4_ and Paclitaxel (PTX) coated with MDA-MB-231 cell membranes (CMs). POx: poly(2-oxazoline)s; IR/SBMA-BSA/GO: sulfobetaine methacrylate (SBMA)-grafted BSA coated GO incorporating IR780; R-O_2_-FA-CHI-SWCNTs: oxygen-carrying tombarthite-modified FA-conjugated chitosan (R-O_2_-FA-CHI)-SWCNT nanocarrier; ANXA5: annexin A5; MWNTs: multi-walled carbon nanotubes

**Fig. 9:**
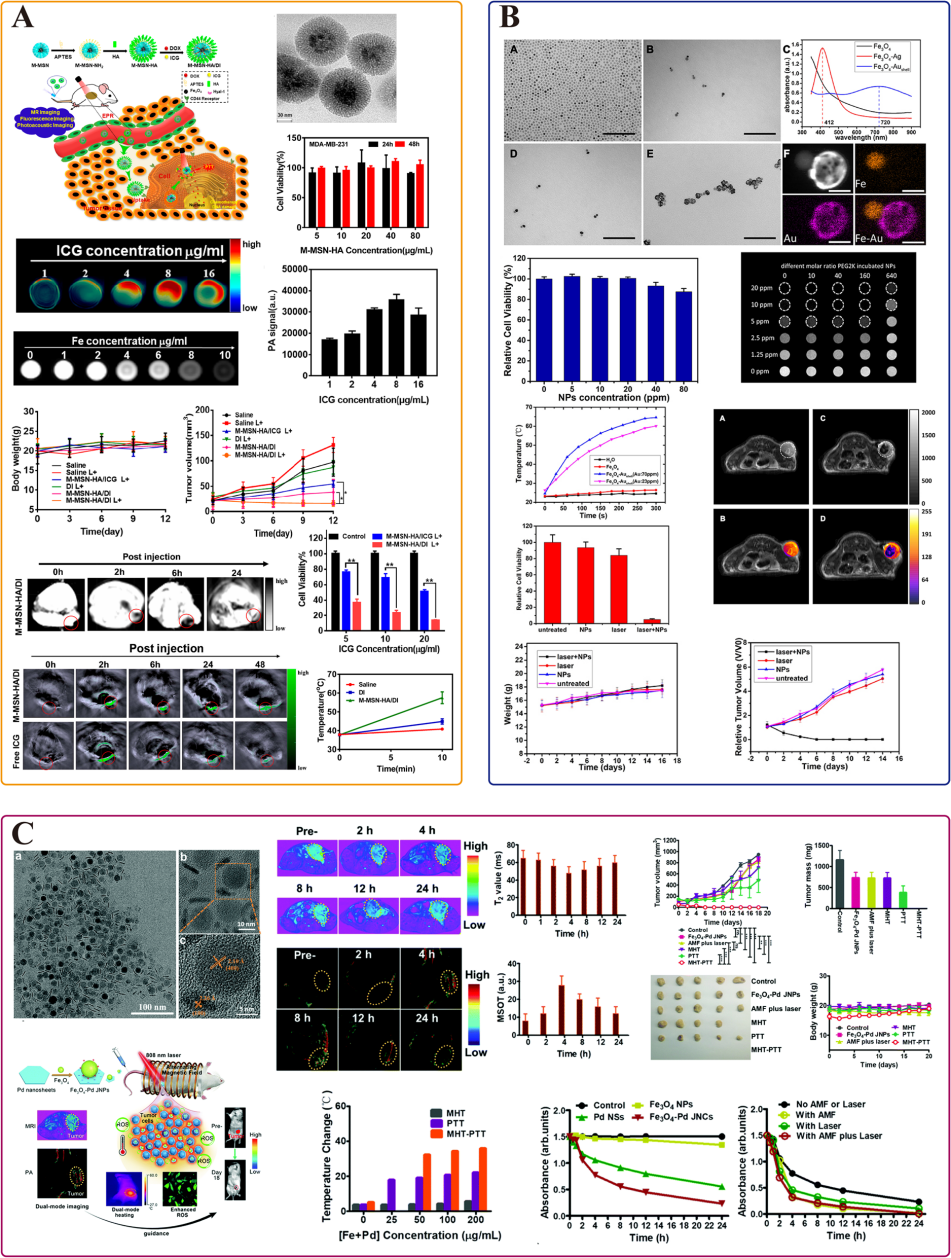
INPs for the local theranostic application of LABC. **A** M-MSN/HA/DI; **B** Fe_3_O_4_–Au_shell_–PEG160 NPs; **C** Fe_3_O_4_–Pd JNPs. **A** Is adapted with permission from [235], copyright 2020 Expert Opinion on Drug Delivery. **B** Is adapted with permission from [241], copyright 2021 International Journal of Nanomedicine. **C** Is adapted with permission from [244], copyright 2019 Nanoscale Horizons

One of the most common functions of INPs is that they carry drugs and are easy to modify. Different functions can be performed by screening and loading suitable drug molecules and by functionalization with different ligands. Among the INPs, MSNs with rich porous structure, large surface area, high biocompatibility, and adjustable surface chemistry are widely used as multifunctional design platforms [234]. Li and colleagues constructed an HA-coated mesoporous silica-coated Fe_3_O_4_ nanoparticles (M-MSN/HA/DI)-based versatile nanoplatforms for the co-delivery of DOX and ICG into MDA-MB-231 breast cancer cells to perform T_2_ MR/FL/PA imaging and chemo/PTT capabilities (Fig. 9A) [235]. In this work, Fe_3_O_4_ NPs play the MRI contrast agent role, whereas ICG, with strong NIR absorption, endows the nanoplatform with PTT and PA/FLI capabilities [236–239]. It is worth noting that M-MSN/HA/DI achieves pH and hyaluronidase-dependent release of DOX, which can reduce the side effects on other organs [240]. After the 808-nm irradiation, the cell activity decreased to 14.1%, depicting an apparent synergistic effect, and the tumor growth was inhibited outstandingly upon combination therapy. For different patients with LABC, INPs represented by MSNs can be modified according to specific needs to play a functional therapeutic effect, which is expected to achieve an image-guided individualized treatment mode.

In addition, the imaging or therapeutic function based on the characteristics of INPs itself cannot be ignored. In another study, Xun et al. designed Fe_3_O_4_–Au_shell_ Janus NPs coated with PEG (Fe_3_O_4_–Au_shell_–PEG160 NPs) to perform synergistic theranostics of MRI and PTT of breast cancer (Fig. 9B) [241]. Fe_3_O_4_ NPs are the most widely studied and applied for MRI, whereas Au_shell_ NPs are widely used PTA with high photothermal conversion efficiency and favorable biocompatibility [242, 243]. After the irradiation of 808-nm NIR (0.65 W/cm^2^, 5 min), the temperature rose to 52.5 °C, and only a few cells (5.1 ± 0.8%) were alive, which indicated a remarkable PTT effect. In addition, in the 4T1 breast cancer mice model, the tumor temperature could rise to 54.6 °C under NIR irradiation, and the tumor ablation was achieved within 6 days of treatment. Moreover, after intratumoral administration of Fe_3_O_4_–Au_shell_–PEG160 NPs, a significant reduction (60.6%) of T_2_WI signal intensity was observed, suggesting its excellent potential as an MRI T_2_ contrast agent.

Based on the AMF response ability of Fe_3_O_4_ NPs itself, MHT can also be introduced to achieve synergistic therapeutic effects. Ma and colleagues constructed the multifunctional Fe_3_O_4_–Pd Janus NPs (Fe_3_O_4_–Pd JNPs) to synergistically facilitate magnetic and NIR hyperthermia, along with enhanced ROS production for breast cancer treatment (Fig. 9C) [244]. Fe_3_O_4_ NPs demonstrated excellent responsiveness to AMF and laser and have been extensively studied and utilized in PTT, MHT, and MRI [245, 246]. Pd nanosheets (Pd NSs) have excellent NIR absorption and photothermal conversion ability, showcasing tremendous application potential for PTT [247]. Moreover, leveraging the Fenton reaction mediated by iron nanomaterials and catalytic properties of Pd NSs in an acidic environment, the nanoplatform could react with H_2_O_2_ in tumor cells to produce hydroxyl radicals, one type of ROS, to kill tumor cells by inducing apoptosis [247–249]. In their study, a synergistic amplification strategy of heating and ROS generation was realized under the action of AMF and NIR. In addition, this synergistic strategy achieved complete 4T1 in situ breast tumor suppression, exhibiting outstanding anti-tumor effects. In addition, due to the inherent characteristics of the Fe_3_O_4_–Pd Janus NPs, they also show potential for imaging applications. As a negative T_2_ MR contrast agent, Fe_3_O_4_–Pd Janus NPs showed a dose-dependent darkening effect. At the same time, the intensity of the PA signal increased linearly with Pd concentration, indicating the potential for MR/PAI application. This design makes rational use of the physical and chemical properties of INPs and the tumor microenvironment, providing a different perspective for treating LABC.

The continuous advancement in the research and development of INPs has provided a platform for the local diagnosis and treatment of LABC. Their load capacity can be used to optimize traditional drugs’ shortcomings or develop novel therapeutic strategies based on their inherent properties, such as laser or AMF-responsive ability. These satisfactory features open exciting possibilities to overcome therapeutic challenges in managing LABC.

## INPs under clinical trials for breast cancer diagnosis and treatment

Recent years have witnessed an increase in the number of nanoparticles undergoing clinical trials. As of 2024, Clinicaltrials.gov lists 90 trials filtered for the condition “breast cancer” and the search term “nanoparticles” [278]. There are 12 trials of INPs in breast cancer diagnosis and treatment (Table 5). The majority applications of these trials are focused on lymph node detection, including six trials based on SPIONs (NCT05161507, NCT06104371, NCT05625698, NCT04722692, NCT05985551, and NCT05359783) and three trials based on carbon nanoparticles (NCT04951245, NCT04482803, and NCT03355261). For systemic anticancer therapy, there are two trials based on carbon nanoparticles (NCT06048367) and CdS/ZnS core–shell type QDs (NCT04138342). For local treatment, there is one trial based on AGuIX gadolinium-based nanoparticles (NCT04899908) as a radiosensitizer for improving the efficacy of radiotherapy. Although the progress of inorganic nanomaterials in clinical transformation is not as rapid as fundamental research, the increasing number of clinical trials shows the urgent clinical demand and promising application prospects.

**Table 5:** INPs under clinical trials for breast cancer diagnosis and treatment

Inorganic nanoplatform	Application	NCT number	Study title	Study type	Phase
Carbon nanoparticles	Lymph node detection	NCT04951245	Ultrasound-assisted CNSs mapping versus dual-tracer-guided sentinel lymph node biopsy	Interventional	Phase 3
SPIONs	Lymph node detection	NCT05161507	Magseed and magtrace localization for breast cancer	Observational	Not applicable
Carbon nanoparticles	Systemic therapy	NCT06048367	Carbon nanoparticle-loaded iron [CNSI-Fe(II)] in the treatment of advanced solid tumor	Interventional	Phase 1
Carbon nanoparticles	Lymph node detection	NCT04482803	Targeted biopsy of carbon nanoparticles labelled axillary node for cN + breast cancer	Interventional	Not applicable
CdS/ZnS core–shell type QDs	Systemic therapy	NCT04138342	Topical fluorescent nanoparticles conjugated somatostatin analog for suppression and bioimaging breast cancer	Interventional	Phase 1
Carbon nanoparticles	Lymph node detection	NCT03355261	Positive node traced before neoadjuvant chemotherapy (NAC)	Interventional	Not applicable
SPIONs	Lymph node detection	NCT06104371	Lymph node identification using magtrace and magseed before chemotherapy	Observational	Not applicable
SPIONs	Lymph node detection	NCT05625698	Premarking of axillary nodes before start of neoadjuvant chemotherapy using magnetic approach	Interventional	Not applicable
SPIONs	Lymph node detection	NCT04722692	Delayed sentinel lymph node biopsy in ductal cancer in situ	Interventional	Phase 3
AGuIX gadolinium-based nanoparticles	Radiotherapy	NCT04899908	Stereotactic brain-directed radiation with or without aguix gadolinium-based nanoparticles in brain metastases	Interventional	Phase 2
SPIONs	Lymph node detection	NCT05985551	Delayed SLND for patients with breast cancer undergoing primary systemic treatment	Observational	Not applicable
SPIONs	Lymph node detection	NCT05359783	Sentinel node localization and staging with low dose superparamagnetic iron oxide	Interventional	Phase 1, 2

The stage of a clinical trial studying a drug or biological product, based on definitions developed by the U.S. Food and Drug Administration (FDA). The phase is based on the study's objective, the number of participants, and other characteristics. There are five phases: early Phase 1 (formerly listed as Phase 0), Phase 1, Phase 2, Phase 3, and Phase 4. Not applicable is used to describe trials without FDA-defined phases, including trials of devices or behavioral interventions

## Conclusion and future perspectives

This review extensively discusses the advances in the development of INPs in the local treatment and diagnosis of breast cancer, which has been a hotspot over the past decade. Also, it provides inspiring optimism for overcoming the treatment and diagnostic barriers of inoperable LABC.

In the face of inoperable LABC, it is critical to develop minimally invasive control options to reduce the stage of LABC. Compared with deep tumors, multiple physical stimulation techniques such as phototherapy, radiotherapy, and SDT are more applicable for the treatment of superficial types of breast cancer. The introduction of INPs has realized the sensitization effect of tumor cells to these treatment regimens and ultimately enhanced the therapeutic effect. Meanwhile, INPs with long half-life and high biocompatibility ensure the effective and continuous role of the efficacy and overcome the side effects and defects of traditional radiosensitizers, PSs, sonosensitizers, and these intermediums. Meanwhile, MHT based on the AMF response characteristics of magnetic nanomaterials can also achieve image-guided treatment mode in combination with MRI. In future studies, the synergistic application mode of various local treatment schemes must be further explored to develop alternative therapies and regents suitable for LABC. For instance, combining the existing gel technology to prepare biosafe dressing products is suggested. Furthermore, according to different breast cancer types, INPs can be specifically targeted by utilizing presenting biomarkers and screening proper targeting groups. This can effectively improve treatment efficiency while reducing normal tissue damage, thus realizing individualized local treatment more accurately.

In the diagnosis of breast cancer, although MRI, CT/PET, PAI, FLI, and SERS provide multidimensional diagnostic information, they each have inherent limitations. Despite its ability to offer high-resolution imaging, MRI poses challenges due to its long-lasting imaging time, potential side effects from conventional contrast agents and the risk of false positives. Similarly, CT/PET provides valuable anatomical and functional data but is constrained by spatial resolution and radiation risks. Although PAI and FLI excel in providing high-resolution images, the stability of their fluorescent signals remains a concern. Although SERS technology has shown remarkable success in laboratory studies, further clinical research is required to establish its repeatability. As novel excellent contrast agents, INPs can realize the real-time treatment detection of tumors and achieve multimodal diagnosis and treatment mode, which is expected to improve the accuracy, sensitivity, and specificity of existing diagnostic strategies to the next generation.

Several important issues must be addressed before nanomaterials can be transformed into clinical applications. Although the mammary gland is a superficial accessory organ, it is also necessary to consider the intrinsic toxicity of the INPs. Elements with low intrinsic toxicity should be recommended as far as possible while constructing inorganic nanoplatforms, whereas befitting modification methods should also be employed to cover such metal cores. Various slow-release methods are also proposed to control the amount of nanomaterial systems. In addition, the following influencing factors, such as particle size and shape, surface ligands, and electric charge, are indispensable. These will be most crucial in influencing the pathway to enter cells and the interactions with biological systems. Moreover, the distribution and metabolism of INPs in vivo still require further observation to monitor the long-term toxicity and systematically assess the impact on the organism's function. Moreover, the tumor microenvironment and the characteristics of different tumor molecular phenotypes should also be considered while deciding on reasonable INPs-based nanoplatforms.

In summary, the issues of the clinical status of LABC diagnosis and treatment have prompted urgent demands for a new generation of nanomaterials. At present, the research of INPs primarily focuses on the establishment of various types of breast cancer tumor models. In contrast, the actual exploration of clinical applications on different stages of breast cancer progression (such as early breast cancer, local progression, etc.) is relatively lacking. Nevertheless, it is undeniable that the development of multifunctional INPs provides novel insights into treating inoperable LABC. With technological innovation, the multifunctional nanoplatform based on INPs is also expected to improve the status quo of treatment and diagnosis of inoperable LABC, benefiting patients.

## Data Availability

No datasets were generated or analysed during the current study.

## References

[1] Siegel RL, Miller KD, Wagle NS, Jemal A (2023). Cancer statistics, 2023. CA Cancer J Clin.

[2] Giaquinto AN, Sung H, Miller KD, Kramer JL, Newman LA, Minihan A (2022). Breast cancer statistics, 2022. CA Cancer J Clin.

[3] Benitez Fuentes JD, Morgan E, de Luna AA, Mafra A, Shah R, Giusti F (2024). Global stage distribution of breast cancer at diagnosis: a systematic review and meta-analysis. JAMA Oncol.

[4] Aebi S, Karlsson P, Wapnir IL (2022). Locally advanced breast cancer. Breast.

[5] Sung H, Ferlay J, Siegel RL, Laversanne M, Soerjomataram I, Jemal A (2021). Global cancer statistics 2020: GLOBOCAN estimates of incidence and mortality worldwide for 36 cancers in 185 countries. CA Cancer J Clin.

[6] Tryfonidis K, Senkus E, Cardoso MJ, Cardoso F (2015). Management of locally advanced breast cancer-perspectives and future directions. Nat Rev Clin Oncol.

[7] Yalcin B (2013). Overview on locally advanced breast cancer: defining, epidemiology, and overview on neoadjuvant therapy. Exp Oncol.

[8] Newman LA (2009). Epidemiology of locally advanced breast cancer. Semin Radiat Oncol.

[9] Maajani K, Jalali A, Alipour S, Khodadost M, Tohidinik HR, Yazdani K (2019). The global and regional survival rate of women with breast cancer: a systematic review and meta-analysis. Clin Breast Cancer.

[10] Loibl S, Poortmans P, Morrow M, Denkert C, Curigliano G (2021). Breast cancer. Lancet.

[11] Afzal M, Ameeduzzafar, Alharbi KS, Alruwaili NK, Al-Abassi FA, Al-Malki AAL (2021). Nanomedicine in treatment of breast cancer—a challenge to conventional therapy. Semin Cancer Biol.

[12] Waks AG, Winer EP (2019). Breast cancer treatment: a review. JAMA.

[13] Wang M, Hou L, Chen M, Zhou Y, Liang Y, Wang S (2017). Neoadjuvant chemotherapy creates surgery opportunities for inoperable locally advanced breast cancer. Sci Rep.

[14] Eisenhauer EA, Therasse P, Bogaerts J, Schwartz LH, Sargent D, Ford R (2009). New response evaluation criteria in solid tumours: revised RECIST guideline (version 1.1). Eur J Cancer.

[15] Thill M, Kolberg-Liedtke C, Albert US, Banys-Paluchowski M, Bauerfeind I, Blohmer JU (2023). AGO recommendations for the diagnosis and treatment of patients with locally advanced and metastatic breast cancer: update 2023. Breast Care.

[16] Allweis TM, Hermann N, Berenstein-Molho R, Guindy M (2021). Personalized screening for breast cancer: rationale, present practices, and future directions. Ann Surg Oncol.

[17] Shi Z, Huang X, Cheng Z, Xu Z, Lin H, Liu C (2023). MRI-based quantification of intratumoral heterogeneity for predicting treatment response to neoadjuvant chemotherapy in breast cancer. Radiology.

[18] Pötsch N, Vatteroni G, Clauser P, Helbich TH, Baltzer PAT (2022). Contrast-enhanced mammography versus contrast-enhanced breast MRI: a systematic review and meta-analysis. Radiology.

[19] Ulaner GA (2019). PET/CT for patients with breast cancer: where is the clinical impact?. Am J Roentgenol.

[20] Mann RM, Hooley R, Barr RG, Moy L (2020). Novel approaches to screening for breast cancer. Radiology.

[21] Kanda MH, da Costa Vieira RA, Lima J, Paiva CE, de Araujo RLC (2020). Late locoregional complications associated with adjuvant radiotherapy in the treatment of breast cancer: systematic review and meta-analysis. J Surg Oncol.

[22] Abdo J, Ortman H, Rodriguez N, Tillman R, Riordan EO, Seydel A (2023). Quality of life issues following breast cancer treatment. Surg Clin N Am.

[23] Pei Z, Lei H, Cheng L (2023). Bioactive inorganic nanomaterials for cancer theranostics. Chem Soc Rev.

[24] Owens TC, Anton N, Attia MF (2023). CT and X-ray contrast agents: current clinical challenges and the future of contrast. Acta Biomater.

[25] Ni D, Bu W, Ehlerding EB, Cai W, Shi J (2017). Engineering of inorganic nanoparticles as magnetic resonance imaging contrast agents. Chem Soc Rev.

[26] Fu Q, Zhu R, Song J, Yang H, Chen X (2019). Photoacoustic imaging: contrast agents and their biomedical applications. Adv Mater.

[27] Li B, Zhao M, Lin J, Huang P, Chen X (2022). Management of fluorescent organic/inorganic nanohybrids for biomedical applications in the NIR-II region. Chem Soc Rev.

[28] Chauhan P, Bhargava A, Kumari R, Ratre P, Tiwari R, Kumar Srivastava R (2022). Surface-enhanced Raman scattering biosensors for detection of oncomiRs in breast cancer. Drug Discov Today.

[29] Li X, Lovell JF, Yoon J, Chen X (2020). Clinical development and potential of photothermal and photodynamic therapies for cancer. Nat Rev Clin Oncol.

[30] Kaur T, Sharma D (2022). Expansion of thermometry in magnetic hyperthermia cancer therapy: antecedence and aftermath. Nanomedicine.

[31] Pei Z, Chen S, Ding L, Liu J, Cui X, Li F (2022). Current perspectives and trend of nanomedicine in cancer: a review and bibliometric analysis. J Control Release.

[32] Freitas LF, Ferreira AH, Thipe VC, Varca GHC, Lima CSA, Batista JGS (2021). The state of the art of theranostic nanomaterials for lung, breast, and prostate cancers. Nanomaterials.

[33] Ge XL, Huang B, Zhang ZL, Liu X, He M, Yu Z (2019). Glucose-functionalized near-infrared Ag(2)Se quantum dots with renal excretion ability for long-term in vivo tumor imaging. J Mater Chem B.

[34] Wen S, Wang W, Liu R, He P (2020). Amylase-protected Ag nanodots for in vivo fluorescence imaging and photodynamic therapy of tumors. Int J Nanomed.

[35] Wang F, Qu L, Ren F, Baghdasaryan A, Jiang Y, Hsu R (2022). High-precision tumor resection down to few-cell level guided by NIR-IIb molecular fluorescence imaging. Proc Natl Acad Sci USA.

[36] Ramesh K, Truong A, Wang Y, Rusckowski M, Gkikas M (2022). Ligand-specific nano-contrast agents promote enhanced breast cancer CT detection at 0.5 mg Au. Int J Mol Sci.

[37] Kwon J, Jun SW, Choi SI, Mao X, Kim J, Koh EK (2019). FeSe quantum dots for in vivo multiphoton biomedical imaging. Sci Adv.

[38] Pascual L, Cerqueira-Coutinho C, García-Fernández A, de Luis B, Bernardes ES, Albernaz MS (2017). MUC1 aptamer-capped mesoporous silica nanoparticles for controlled drug delivery and radio-imaging applications. Nanomed Nanotechnol Biol Med.

[39] Dong L, Li W, Yu L, Sun L, Chen Y, Hong G (2020). Ultrasmall Ag_2_Te quantum dots with rapid clearance for amplified computed tomography imaging and augmented photonic tumor hyperthermia. ACS Appl Mater Interfaces.

[40] Chan HN, Ho SL, He D, Li HW (2020). Direct and sensitive detection of circulating miRNA in human serum by ligase-mediated amplification. Talanta.

[41] Bahari D, Babamiri B, Salimi A (2021). Ultrasensitive molecularly imprinted fluorescence sensor for simultaneous determination of CA125 and CA15-3 in human serum and OVCAR-3 and MCF-7 cells lines using Cd and Ni nanoclusters as new emitters. Anal Bioanal Chem.

[42] Feng E, Zheng T, He X, Chen J, Tian Y (2018). A novel ternary heterostructure with dramatic SERS activity for evaluation of PD-L1 expression at the single-cell level. Sci Adv.

[43] Du Y, Liu X, Liang Q, Liang XJ, Tian J (2019). Optimization and design of magnetic ferrite nanoparticles with uniform tumor distribution for highly sensitive MRI/MPI performance and improved magnetic hyperthermia therapy. Nano Lett.

[44] Guo Y, Wang XY, Chen YL, Liu FQ, Tan MX, Ao M (2018). A light-controllable specific drug delivery nanoplatform for targeted bimodal imaging-guided photothermal/chemo synergistic cancer therapy. Acta Biomater.

[45] Xu F, Li X, Chen H, Jian M, Sun Y, Liu G (2020). Synthesis of heteronanostructures for multimodality molecular imaging-guided photothermal therapy. J Mater Chem B.

[46] Yang J, Su H, Sun W, Cai J, Liu S, Chai Y (2018). Dual chemodrug-loaded single-walled carbon nanohorns for multimodal imaging-guided chemo-photothermal therapy of tumors and lung metastases. Theranostics.

[47] Dong Y, Liu Y, Tu Y, Yuan Y, Wang J (2023). AIEgens cross-linked iron oxide nanoparticles synchronously amplify bimodal imaging signals in situ by tumor acidity-mediated click reaction. Angew Chem Int Ed Engl.

[48] Coates AS, Winer EP, Goldhirsch A, Gelber RD, Gnant M, Piccart-Gebhart M (2015). Tailoring therapies—improving the management of early breast cancer: St Gallen international expert consensus on the primary therapy of early breast cancer 2015. Ann Oncol.

[49] Gilbert FJ, Pinker-Domenig K (2019). Diagnosis and staging of breast cancer: when and how to use mammography, tomosynthesis, ultrasound, contrast-enhanced mammography, and magnetic resonance imaging. Diseases of the chest, breast, heart and vessels 2019–2022: diagnostic and interventional imaging.

[50] Wan L, Chen Z, Deng Y, Liao T, Kuang Y, Liu J (2020). A novel intratumoral pH/redox-dual-responsive nanoplatform for cancer MR imaging and therapy. J Colloid Interface Sci.

[51] Xie L, Jin W, Zuo X, Ji S, Nan W, Chen H (2020). Construction of small-sized superparamagnetic Janus nanoparticles and their application in cancer combined chemotherapy and magnetic hyperthermia. Biomater Sci.

[52] Zavvar T, Babaei M, Abnous K, Taghdisi SM, Nekooei S, Ramezani M (2020). Synthesis of multimodal polymersomes for targeted drug delivery and MR/fluorescence imaging in metastatic breast cancer model. Int J Pharm.

[53] Chen S, Zhang Q, Sun H, Zheng Y, Chen Q, Luo Y (2020). A cation exchange strategy to construct a targeting nanoprobe for enhanced T_1_-weighted MR imaging of tumors. J Mater Chem B.

[54] Nieves LM, Hsu JC, Lau KC, Maidment ADA, Cormode DP (2021). Silver telluride nanoparticles as biocompatible and enhanced contrast agents for X-ray imaging: an in vivo breast cancer screening study. Nanoscale.

[55] Prasad R, Agawane SB, Chauhan DS, Srivastava R, Selvaraj K (2018). In vivo examination of folic acid-conjugated gold-silica nanohybrids as contrast agents for localized tumor diagnosis and biodistribution. Bioconjug Chem.

[56] Wang R, Deng J, He D, Yang E, Yang W, Shi D (2019). PEGylated hollow gold nanoparticles for combined X-ray radiation and photothermal therapy in vitro and enhanced CT imaging in vivo. Nanomed Nanotechnol Biol Med.

[57] Liu Z, Liang Y, Cao W, Gao W, Tang B (2021). Proximity-induced hybridization chain reaction-based photoacoustic imaging system for amplified visualization protein-specific glycosylation in mice. Anal Chem.

[58] Cao W, Gao W, Liu Z, Hao W, Li X, Sun Y (2018). Visualizing miR-155 to monitor breast tumorigenesis and response to chemotherapeutic drugs by a self-assembled photoacoustic nanoprobe. Anal Chem.

[59] Dai X, Zhao X, Liu Y, Chen B, Ding X, Zhao N (2021). Controlled synthesis and surface engineering of janus chitosan-gold nanoparticles for photoacoustic imaging-guided synergistic gene/photothermal therapy. Small.

[60] Xu L, Du J, Wan C, Zhang Y, Xie S, Li H (2018). Ultrasound molecular imaging of breast cancer in MCF-7 orthotopic mice using gold nanoshelled poly(lactic-co-glycolic acid) nanocapsules: a novel dual-targeted ultrasound contrast agent. Int J Nanomed.

[61] Cui G, He P, Yu L, Wen C, Xie X, Yao G (2020). Oxygen self-enriched nanoplatform combined with US imaging and chemo/photothermal therapy for breast cancer. Nanomed Nanotechnol Biol Med.

[62] Ou YC, Webb JA, O'Brien CM, Pence IJ, Lin EC, Paul EP (2018). Diagnosis of immunomarkers in vivo via multiplexed surface enhanced Raman spectroscopy with gold nanostars. Nanoscale.

[63] Wei Q, He J, Wang S, Hua S, Qi Y, Li F (2021). Low-dose X-ray enhanced tumor accumulation of theranostic nanoparticles for high-performance bimodal imaging-guided photothermal therapy. J Nanobiotechnol.

[64] Kang S, Kang K, Chae A, Kim YK, Jang H, Min DH (2019). Fucoidan-coated coral-like Pt nanoparticles for computed tomography-guided highly enhanced synergistic anticancer effect against drug-resistant breast cancer cells. Nanoscale.

[65] Hajiramezanali M, Atyabi F, Mosayebnia M, Akhlaghi M, Geramifar P, Jalilian AR (2019). ^68^Ga-radiolabeled bombesin-conjugated to trimethyl chitosan-coated superparamagnetic nanoparticles for molecular imaging: preparation, characterization and biological evaluation. Int J Nanomed.

[66] Hu P, Shang L, Chen J, Chen X, Chen C, Hong W (2020). A nanometer-sized protease inhibitor for precise cancer diagnosis and treatment. J Mater Chem B.

[67] Turnbull LW (2009). Dynamic contrast-enhanced MRI in the diagnosis and management of breast cancer. NMR Biomed.

[68] Chitambar CR (2010). Medical applications and toxicities of gallium compounds. Int J Environ Res Public Health.

[69] Maturi M, Locatelli E, Monaco I, Comes FM (2019). Current concepts in nanostructured contrast media development for in vivo photoacoustic imaging. Biomater Sci.

[70] Chee HL, Gan CRR, Ng M, Low L, Fernig DG, Bhakoo KK (2018). Biocompatible peptide-coated ultrasmall superparamagnetic iron oxide nanoparticles for in vivo contrast-enhanced magnetic resonance imaging. ACS Nano.

[71] Li L, Wu C, Pan L, Li X, Kuang A, Cai H (2019). Bombesin-functionalized superparamagnetic iron oxide nanoparticles for dual-modality MR/NIRFI in mouse models of breast cancer. Int J Nanomed.

[72] Du J, Zhang Y, Jin Z, Wu H, Cang J, Shen Y (2020). Targeted NIRF/MR dual-mode imaging of breast cancer brain metastasis using BRBP1-functionalized ultra-small iron oxide nanoparticles. Mater Sci Eng C Mater Biol Appl.

[73] Dayes IS, Metser U, Hodgson N, Parpia S, Eisen AF, George R (2023). Impact of ^18^F-labeled fluorodeoxyglucose positron emission tomography-computed tomography versus conventional staging in patients with locally advanced breast cancer. J Clin Oncol.

[74] Arnaout A, Varela NP, Allarakhia M, Grimard L, Hey A, Lau J (2020). Baseline staging imaging for distant metastasis in women with stages I, II, and III breast cancer. Curr Oncol.

[75] Nie L, Chen X (2014). Structural and functional photoacoustic molecular tomography aided by emerging contrast agents. Chem Soc Rev.

[76] Wang S, Lin J, Wang T, Chen X, Huang P (2016). Recent advances in photoacoustic imaging for deep-tissue biomedical applications. Theranostics.

[77] Hu X, Sun X, Liu X, Xu HD, Yang L, Liu S (2023). Enhanced photoacoustic imaging of urokinase-type plasminogen activator activity in tumors. Anal Chem.

[78] Nagaoka R, Tabata T, Yoshizawa S, Umemura SI, Saijo Y (2018). Visualization of murine lymph vessels using photoacoustic imaging with contrast agents. Photoacoustics.

[79] Yildiz Potter I, Yeritsyan D, Mahar S, Wu J, Nazarian A, Vaziri A (2023). Automated bone tumor segmentation and classification as benign or malignant using computed tomographic imaging. J Digit Imaging.

[80] Yang Y, Liu Q, Dai T, Zhang H (2023). Automatic detection of benign/malignant tumor in breast ultrasound images using optimal features. Curr Med Imaging.

[81] Zhang W, Zhang CC, Wang XY, Li L, Chen QQ, Liu WW (2020). Light-responsive core-shell nanoplatform for bimodal imaging-guided photothermal therapy-primed cancer immunotherapy. ACS Appl Mater Interfaces.

[82] Kawelah MR, Han S, Atila Dincer C, Jeon J, Brisola J, Hussain AF (2024). Antibody-conjugated polymersomes with encapsulated indocyanine green J-aggregates and high near-infrared absorption for molecular photoacoustic cancer imaging. ACS Appl Mater Interfaces.

[83] Adhami M, Haghdoost AA, Sadeghi B, Malekpour AR (2018). Candidate miRNAs in human breast cancer biomarkers: a systematic review. Breast Cancer.

[84] Liu L, Xiong H, Wang X, Jiang H (2024). Gold nanomaterials: important vectors in biosensing of breast cancer biomarkers. Anal Bioanal Chem.

[85] Tarighati E, Keivan H, Mahani H (2023). A review of prognostic and predictive biomarkers in breast cancer. Clin Exp Med.

[86] Xu X, Li H, Li K, Zeng Q, Liu Y, Zeng Y (2019). A photo-triggered conjugation approach for attaching RGD ligands to biodegradable mesoporous silica nanoparticles for the tumor fluorescent imaging. Nanomed Nanotechnol Biol Med.

[87] Cao YC, Jin R, Mirkin CA (2002). Nanoparticles with Raman spectroscopic fingerprints for DNA and RNA detection. Science.

[88] Le Ru EC, Etchegoin PG (2012). Single-molecule surface-enhanced Raman spectroscopy. Annu Rev Phys Chem.

[89] Wang X, Chen C, Chen C, Zuo E, Han S, Yang J (2023). Novel SERS biosensor for rapid detection of breast cancer based on Ag_2_O-Ag-PSi nanochips. Spectrochim Acta A Mol Biomol Spectrosc.

[90] Lee JU, Kim WH, Lee HS, Park KH, Sim SJ (2019). Quantitative and specific detection of exosomal miRNAs for accurate diagnosis of breast cancer using a surface-enhanced Raman scattering sensor based on plasmonic head-flocked gold nanopillars. Small.

[91] Murali VP, Karunakaran V, Murali M, Lekshmi A, Kottarathil S, Deepika S (2023). A clinically feasible diagnostic spectro-histology built on SERS-nanotags for multiplex detection and grading of breast cancer biomarkers. Biosens Bioelectron.

[92] Zheng Z, Wu L, Li L, Zong S, Wang Z, Cui Y (2018). Simultaneous and highly sensitive detection of multiple breast cancer biomarkers in real samples using a SERS microfluidic chip. Talanta.

[93] Cheung KL, Graves CR, Robertson JF (2000). Tumour marker measurements in the diagnosis and monitoring of breast cancer. Cancer Treat Rev.

[94] Berghuis AMS, Koffijberg H, Prakash J, Terstappen LW, IJzerman MJ (2017). Detecting blood-based biomarkers in metastatic breast cancer: a systematic review of their current status and clinical utility. Int J Mol Sci.

[95] Cani AK, Hayes DF (2024). Breast cancer circulating tumor cells: current clinical applications and future prospects. Clin Chem.

[96] Li H, Wang S, Li X, Cheng C, Shen X, Wang T (2022). Dual-channel detection of breast cancer biomarkers CA15-3 and CEA in human serum using dialysis-silicon nanowire field effect transistor. Int J Nanomed.

[97] Ranjan P, Abubakar Sadique M, Yadav S, Khan R (2022). An electrochemical immunosensor based on gold-graphene oxide nanocomposites with ionic liquid for detecting the breast cancer CD44 biomarker. ACS Appl Mater Interfaces.

[98] Yola ML (2021). Sensitive sandwich-type voltammetric immunosensor for breast cancer biomarker HER2 detection based on gold nanoparticles decorated Cu-MOF and Cu_2_ZnSnS_4_ NPs/Pt/g-C_3_N_4_ composite. Mikrochim Acta.

[99] Eniu A, Salati E, Durigova A (2023). Precision medicine in early breast cancer-beginning of a successful story?. ESMO Open.

[100] Grzywa R, Lupicka-Slowik A, Walczak M, Idzi M, Bobrek K, Boivin S (2014). Highly sensitive detection of cancer antigen 15-3 using novel avian IgY antibodies. Altex.

[101] Ravalli A, Lozzi L, Marrazza G (2016). Micro-flow immunosensor based on thin-film interdigitated gold array microelectrodes for cancer biomarker detection. Curr Drug Deliv.

[102] Pang X, Li J, Zhao Y, Wu D, Zhang Y, Du B (2015). Label-free electrochemiluminescent immunosensor for detection of carcinoembryonic antigen based on nanocomposites of GO/MWCNTs-COOH/Au@CeO_2_. ACS Appl Mater Interfaces.

[103] Saify Nabiabad H, Piri K, Kafrashi F, Afkhami A, Madrakian T (2018). Fabrication of an immunosensor for early and ultrasensitive determination of human tissue plasminogen activator (tPA) in myocardial infraction and breast cancer patients. Anal Bioanal Chem.

[104] Li B, Pan W, Liu C, Guo J, Shen J, Feng J (2020). Homogenous magneto-fluorescent nanosensor for tumor-derived exosome isolation and analysis. ACS Sens.

[105] Wang C, Zhang Y, Tang W, Wang C, Han Y, Qiang L (2021). Ultrasensitive, high-throughput and multiple cancer biomarkers simultaneous detection in serum based on graphene oxide quantum dots integrated microfluidic biosensing platform. Anal Chim Acta.

[106] Perez WI, Soto Y, Ramirez-Vick JE, Melendez E (2015). Nanostructured gold dsDNA sensor for early detection of breast cancer by beta protein 1 (BP_1_). J Electroanal Chem.

[107] You M, Yang S, Tang W, Zhang F, He P (2018). Molecularly imprinted polymers-based electrochemical DNA biosensor for the determination of BRCA-1 amplified by SiO_2_@Ag. Biosens Bioelectron.

[108] Alarfaj NA, El-Tohamy MF, Oraby H (2018). New label-free ultrasensitive electrochemical immunosensor-based Au/MoS_2_/rGO nanocomposites for CA 27–29 breast cancer antigen detection. New J Chem.

[109] Cotchim S, Thavarungkul P, Kanatharana P, Limbut W (2020). Multiplexed label-free electrochemical immunosensor for breast cancer precision medicine. Anal Chim Acta.

[110] Qiao B, Guo Q, Jiang J, Qi Y, Zhang H, He B (2019). An electrochemiluminescent aptasensor for amplified detection of exosomes from breast tumor cells (MCF-7 cells) based on G-quadruplex/hemin DNAzymes. Analyst.

[111] Sha Q, Guan R, Su H, Zhang L, Liu BF, Hu Z (2020). Carbohydrate-protein template synthesized high mannose loading gold nanoclusters: a powerful fluorescence probe for sensitive Concanavalin A detection and specific breast cancer cell imaging. Talanta.

[112] Zhang M, Gao G, Ding Y, Deng C, Xiang J, Wu H (2019). A fluorescent aptasensor for the femtomolar detection of epidermal growth factor receptor-2 based on the proximity of G-rich sequences to Ag nanoclusters. Talanta.

[113] Meng S, Chen R, Xie J, Li J, Cheng J, Xu Y (2021). Surface-enhanced Raman scattering holography chip for rapid, sensitive and multiplexed detection of human breast cancer-associated microRNAs in clinical samples. Biosens Bioelectron.

[114] Ebrahimi A, Nikokar I, Zokaei M, Bozorgzadeh E (2018). Design, development and evaluation of microRNA-199a-5p detecting electrochemical nanobiosensor with diagnostic application in triple negative breast cancer. Talanta.

[115] Si Y, Xu L, Deng T, Zheng J, Li J (2020). Catalytic hairpin self-assembly-based SERS sensor array for the simultaneous measurement of multiple cancer-associated miRNAs. ACS Sens.

[116] Hakimian F, Ghourchian H (2020). Ultrasensitive electrochemical biosensor for detection of microRNA-155 as a breast cancer risk factor. Anal Chim Acta.

[117] Zouari M, Campuzano S, Pingarrón JM, Raouafi N (2020). Femtomolar direct voltammetric determination of circulating miRNAs in sera of cancer patients using an enzymeless biosensor. Anal Chim Acta.

[118] Wang HN, Crawford BM, Fales AM, Bowie ML, Seewaldt VL, Vo-Dinh T (2016). Multiplexed detection of microRNA biomarkers using SERS-based inverse molecular sentinel (iMS) nanoprobes. J Phys Chem C Nanomater Interfaces.

[119] Zhao J, Liu C, Li Y, Ma Y, Deng J, Li L (2020). Thermophoretic detection of exosomal microRNAs by nanoflares. J Am Chem Soc.

[120] Han Y, Qiang L, Gao Y, Gao J, He Q, Liu H (2021). Large-area surface-enhanced Raman spectroscopy substrate by hybrid porous GaN with Au/Ag for breast cancer miRNA detection. Appl Surf Sci.

[121] Ranjbari S, Rezayi M, Arefinia R, Aghaee-Bakhtiari SH, Hatamluyi B, Pasdar A (2023). A novel electrochemical biosensor based on signal amplification of Au HFGNs/PnBA-MXene nanocomposite for the detection of miRNA-122 as a biomarker of breast cancer. Talanta.

[122] Zhang H, Fu C, Yi Y, Zhou X, Zhou C, Ying G (2018). A magnetic-based SERS approach for highly sensitive and reproducible detection of cancer-related serum microRNAs. Anal Methods.

[123] Song C, Zhang J, Jiang X, Gan H, Zhu Y, Peng Q (2021). SPR/SERS dual-mode plasmonic biosensor via catalytic hairpin assembly-induced AuNP network. Biosens Bioelectron.

[124] Gupta P, Bharti A, Kaur N, Singh S, Prabhakar N (2018). An electrochemical aptasensor based on gold nanoparticles and graphene oxide doped poly(3,4-ethylenedioxythiophene) nanocomposite for detection of MUC1. J Electroanal Chem.

[125] Israel BB, Tilghman SL, Parker-Lemieux K, Payton-Stewart F (2018). Phytochemicals: current strategies for treating breast cancer. Oncol Lett.

[126] Dent RA, Cescon DW, Bachelot T, Jung KH, Shao ZM, Saji S (2023). TROPION-Breast02: datopotamab deruxtecan for locally recurrent inoperable or metastatic triple-negative breast cancer. Future Oncol.

[127] Moran MS, Haffty BG (2009). Radiation techniques and toxicities for locally advanced breast cancer. Semin Radiat Oncol.

[128] Boateng F, Ngwa W (2019). Delivery of nanoparticle-based radiosensitizers for radiotherapy applications. Int J Mol Sci.

[129] Liu Y, Zhang P, Li F, Jin X, Li J, Chen W (2018). Metal-based nanoenhancers for future radiotherapy: radiosensitizing and synergistic effects on tumor cells. Theranostics.

[130] Wang H, Mu X, He H, Zhang XD (2018). Cancer radiosensitizers. Trends Pharmacol Sci.

[131] Hainfeld JF, Dilmanian FA, Zhong Z, Slatkin DN, Kalef-Ezra JA, Smilowitz HM (2010). Gold nanoparticles enhance the radiation therapy of a murine squamous cell carcinoma. Phys Med Biol.

[132] Liu Y, Liu X, Jin X, He P, Zheng X, Dai Z (2015). The dependence of radiation enhancement effect on the concentration of gold nanoparticles exposed to low- and high-LET radiations. Physica Med.

[133] Herold DM, Das IJ, Stobbe CC, Iyer RV, Chapman JD (2000). Gold microspheres: a selective technique for producing biologically effective dose enhancement. Int J Radiat Biol.

[134] Yook S, Cai Z, Lu Y, Winnik MA, Pignol JP, Reilly RM (2016). Intratumorally injected ^177^Lu-labeled gold nanoparticles: gold nanoseed brachytherapy with application for neoadjuvant treatment of locally advanced breast cancer. J Nuclear Med.

[135] Rajaee A, Wang S, Zhao L, Wang D, Liu Y, Wang J (2019). Multifunction bismuth gadolinium oxide nanoparticles as radiosensitizer in radiation therapy and imaging. Phys Med Biol.

[136] Darfarin G, Salehi R, Alizadeh E, Nasiri Motlagh B, Akbarzadeh A, Farajollahi A (2018). The effect of SiO_2_/Au core-shell nanoparticles on breast cancer cell’s radiotherapy. Artif Cells Nanomed Biotechnol.

[137] Hu H, Zheng S, Hou M, Zhu K, Chen C, Wu Z (2022). Functionalized Au@Cu-Sb-S nanoparticles for spectral CT/photoacoustic imaging-guided synergetic photo-radiotherapy in breast cancer. Int J Nanomed.

[138] Chen F, Zhang XH, Hu XD, Zhang W, Lou ZC, Xie LH (2015). Enhancement of radiotherapy by ceria nanoparticles modified with neogambogic acid in breast cancer cells. Int J Nanomed.

[139] Ji C, Zhao M, Wang C, Liu R, Zhu S, Dong X (2022). Biocompatible tantalum nanoparticles as radiosensitizers for enhancing therapy efficacy in primary tumor and metastatic sentinel lymph nodes. ACS Nano.

[140] Matsudaira H, Ueno AM, Furuno I (1980). Iodine contrast medium sensitizes cultured mammalian cells to X rays but not to gamma rays. Radiat Res.

[141] Kada T (1969). Radiosensitization by potassium iodate and related compounds. Int J Radiat Biol Relat Stud Phys Chem Med.

[142] Yamashita S, Namba H, Nagataki S (1993). Thyroid and radiation. Nihon Naibunpi Gakkai zasshi.

[143] Meng HM, Hu XX, Kong GZ, Yang C, Fu T, Li ZH (2018). Aptamer-functionalized nanoscale metal-organic frameworks for targeted photodynamic therapy. Theranostics.

[144] Cline BL, Jiang W, Lee C, Cao Z, Yang X, Zhan S (2021). Potassium iodide nanoparticles enhance radiotherapy against breast cancer by exploiting the sodium-iodide symporter. ACS Nano.

[145] Esnouf A, Wright PA, Moore JC, Ahmed S (2007). Depth of penetration of an 850nm wavelength low level laser in human skin. Acupunct Electrother Res.

[146] Castano AP, Demidova TN, Hamblin MR (2004). Mechanisms in photodynamic therapy: part one-photosensitizers, photochemistry and cellular localization. Photodiagn Photodyn Ther.

[147] Alamdari SG, Amini M, Jalilzadeh N, Baradaran B, Mohammadzadeh R, Mokhtarzadeh A (2022). Recent advances in nanoparticle-based photothermal therapy for breast cancer. J Control Release.

[148] Zhang XY, Zhang PY (2020). Nanotechnology for multimodality treatment of cancer. Oncol Lett.

[149] Mendes R, Pedrosa P, Lima JC, Fernandes AR, Baptista PV (2017). Photothermal enhancement of chemotherapy in breast cancer by visible irradiation of gold nanoparticles. Sci Rep.

[150] Habash RWY (2018). Therapeutic hyperthermia. Handbook of clinical neurology.

[151] van Straten D, Mashayekhi V, de Bruijn HS, Oliveira S, Robinson DJ (2017). Oncologic photodynamic therapy: basic principles, current clinical status and future directions. Cancers.

[152] Han HS, Choi KY (2021). Advances in nanomaterial-mediated photothermal cancer therapies: toward clinical applications. Biomedicines.

[153] Montaseri H, Kruger CA, Abrahamse H (2021). Inorganic nanoparticles applied for active targeted photodynamic therapy of breast cancer. Pharmaceutics.

[154] Pansare V, Hejazi S, Faenza W, Prud'homme RK (2012). Review of long-wavelength optical and NIR imaging materials: contrast agents, fluorophores and multifunctional nano carriers. Chem Mater.

[155] Leitão MM, de Melo-Diogo D, Alves CG, Lima-Sousa R, Correia IJ (2020). Prototypic heptamethine cyanine incorporating nanomaterials for cancer phototheragnostic. Adv Healthc Mater.

[156] Gai S, Yang G, Yang P, He F, Lin J, Jin D (2018). Recent advances in functional nanomaterials for light-triggered cancer therapy. Chem Mater.

[157] Chen H, Shao L, Li Q, Wang J (2013). Gold nanorods and their plasmonic properties. Chem Soc Rev.

[158] Singh P, Mijakovic I (2021). Advances in gold nanoparticle technology as a tool for diagnostics and treatment of cancer. Expert Rev Mol Diagn.

[159] Hirsch LR, Stafford RJ, Bankson JA, Sershen SR, Rivera B, Price RE (2003). Nanoshell-mediated near-infrared thermal therapy of tumors under magnetic resonance guidance. Proc Natl Acad Sci USA.

[160] Meyers MO, Klauber-Demore N, Ollila DW, Amos KD, Moore DT, Drobish AA (2011). Impact of breast cancer molecular subtypes on locoregional recurrence in patients treated with neoadjuvant chemotherapy for locally advanced breast cancer. Ann Surg Oncol.

[161] Cheng Y, Bao D, Chen X, Wu Y, Wei Y, Wu Z (2021). Microwave-triggered/HSP-targeted gold nano-system for triple-negative breast cancer photothermal therapy. Int J Pharm.

[162] Calderwood SK, Gong J (2016). Heat shock proteins promote cancer: it’s a protection racket. Trends Biochem Sci.

[163] Dudeja V, Vickers SM, Saluja AK (2009). The role of heat shock proteins in gastrointestinal diseases. Gut.

[164] Li M, Li L, Zhan C, Kohane DS (2016). Core-shell nanostars for multimodal therapy and imaging. Theranostics.

[165] Chen Y, Tan C, Zhang H, Wang L (2015). Two-dimensional graphene analogues for biomedical applications. Chem Soc Rev.

[166] Augustine S, Singh J, Srivastava M, Sharma M, Das A, Malhotra BD (2017). Recent advances in carbon based nanosystems for cancer theranostics. Biomater Sci.

[167] Liu Y, Bhattarai P, Dai Z, Chen X (2019). Photothermal therapy and photoacoustic imaging via nanotheranostics in fighting cancer. Chem Soc Rev.

[168] Fan X, Pang W, Feng H, Zhang R, Zhu W, Wang Q (2022). Light-guided tumor diagnosis and therapeutics: from nanoclusters to polyoxometalates. Chin Chem Lett.

[169] Zhou B, Wu Q, Wang M, Hoover A, Wang X, Zhou F (2020). Immunologically modified MnFe_2_O_4_ nanoparticles to synergize photothermal therapy and immunotherapy for cancer treatment. Chem Eng J.

[170] Hashemkhani M, Celikbas E, Khan M, Sennaroglu A, Yagci AH (2023). ALA/Ag_2_S/MnO_2_ hybrid nanoparticles for near-infrared image-guided long-wavelength phototherapy of breast cancer. ACS Biomater Sci Eng.

[171] Li X, Wang Y, Liu T, Zhang Y, Wang C, Xie B (2023). Ultrasmall graphene oxide for combination of enhanced chemotherapy and photothermal therapy of breast cancer. Colloids Surf B.

[172] Hu X, Lu Y, Zhao W, Sun M, Li R, Feng L (2021). A PDA-DTC/Cu-MnO_2_ nanoplatform for MR imaging and multi-therapy for triple-negative breast cancer treatment. Chem Commun.

[173] Yang W, Guo W, Le W, Lv G, Zhang F, Shi L (2016). Albumin-bioinspired Gd:CuS nanotheranostic agent for in vivo photoacoustic/magnetic resonance imaging-guided tumor-targeted photothermal therapy. ACS Nano.

[174] Zhang S, Sun C, Zeng J, Sun Q, Wang G, Wang Y (2016). Ambient aqueous synthesis of ultrasmall PEGylated Cu_2__−__x_ se nanoparticles as a multifunctional theranostic agent for multimodal imaging guided photothermal therapy of cancer. Adv Mater.

[175] Chen H, Song M, Tang J, Hu G, Xu S, Guo Z (2016). Ultrahigh ^19^F loaded Cu_1.75_S nanoprobes for simultaneous ^19^F magnetic resonance imaging and photothermal therapy. ACS Nano.

[176] Wang R, He Z, Cai P, Zhao Y, Gao L, Yang W (2019). Surface-functionalized modified copper sulfide nanoparticles enhance checkpoint blockade tumor immunotherapy by photothermal therapy and antigen capturing. ACS Appl Mater Interfaces.

[177] Tang Y, Tan Y, Lin K, Zhu M (2021). Research progress on polydopamine nanoparticles for tissue engineering. Front Chem.

[178] Ying M, Li Q, Wu J, Jiang Y, Xu Z, Ma M (2021). CuS@BSA-NB2 nanoparticles for HER2-targeted photothermal therapy. Front Pharmacol.

[179] Xu W, Qian J, Hou G, Wang Y, Wang J, Sun T (2018). PEGylated hydrazided gold nanorods for pH-triggered chemo/photodynamic/photothermal triple therapy of breast cancer. Acta Biomater.

[180] Fu B, Dang M, Tao J, Li Y, Tang Y (2020). Mesoporous platinum nanoparticle-based nanoplatforms for combined chemo-photothermal breast cancer therapy. J Colloid Interface Sci.

[181] Heidari Z, Salouti M, Sariri R (2015). Breast cancer photothermal therapy based on gold nanorods targeted by covalently-coupled bombesin peptide. Nanotechnology.

[182] Mendoza-Nava H, Ferro-Flores G, Ramírez FM, Ocampo-García B, Santos-Cuevas C, Azorín-Vega E (2017). Fluorescent, plasmonic, and radiotherapeutic properties of the ^177^Lu-dendrimer-AuNP-folate-bombesin nanoprobe located inside cancer cells. Mol Imaging.

[183] Chiu WJ, Chen YC, Huang CC, Yang L, Yu J, Huang SW (2021). Iron hydroxide/oxide-reduced graphene oxide nanocomposite for dual-modality photodynamic and photothermal therapy in vitro and in vivo. Nanomaterials.

[184] Abedin MR, Umapathi S, Mahendrakar H, Laemthong T, Coleman H, Muchangi D (2018). Polymer coated gold-ferric oxide superparamagnetic nanoparticles for theranostic applications. J Nanobiotechnol.

[185] Deng L, Cai X, Sheng D, Yang Y, Strohm EM, Wang Z (2017). A laser-activated biocompatible theranostic nanoagent for targeted multimodal imaging and photothermal therapy. Theranostics.

[186] Demina PA, Khaydukov KV, Babayeva G, Varaksa PO, Atanova AV, Stepanov ME (2023). Upconversion nanoparticles intercalated in large polymer micelles for tumor imaging and chemo/photothermal therapy. Int J Mol Sci.

[187] Singh P, Haloi P, Singh K, Roy S, Sarkar A (2023). Palladium nanocapsules for photothermal therapy in the near-infrared II biological window. ACS Appl Mater Interfaces.

[188] Poulose AC, Veeranarayanan S, Mohamed MS, Aburto RR, Mitcham T, Bouchard RR (2016). Multifunctional Cu_2–x_ Te nanocubes mediated combination therapy for multi-drug resistant MDA MB 453. Sci Rep.

[189] Ong ZY, Chen S, Nabavi E, Regoutz A, Payne DJ, Elson DS (2017). Multibranched gold nanoparticles with intrinsic LAT-1 targeting capabilities for selective photothermal therapy of breast cancer. ACS Appl Mater Interfaces.

[190] Sears J, Swanner J, Fahrenholtz CD, Snyder C, Rohde M, Levi-Polyachenko N (2021). Combined photothermal and ionizing radiation sensitization of triple-negative breast cancer using triangular silver nanoparticles. Int J Nanomed.

[191] Tang J, Li B, Howard CB, Mahler SM, Thurecht KJ, Wu Y (2019). Multifunctional lipid-coated calcium phosphate nanoplatforms for complete inhibition of large triple negative breast cancer via targeted combined therapy. Biomaterials.

[192] Chen H, Burnett J, Zhang F, Zhang J, Paholak H, Sun D (2014). Highly crystallized iron oxide nanoparticles as effective and biodegradable mediators for photothermal cancer therapy. J Mater Chem B.

[193] Zheng Y, Chen J, Song XR, Chang MQ, Feng W, Huang H (2023). Manganese-enriched photonic/catalytic nanomedicine augments synergistic anti-TNBC photothermal/nanocatalytic/immuno-therapy via activating cGAS-STING pathway. Biomaterials.

[194] Chen S, Huang B, Pei W, Xu Y, Jiang Z, Li J (2019). Magnetically targeted nanoparticles for imaging-guided photothermal therapy of cancer. RSC Adv.

[195] Dobson J, de Queiroz GF, Golding JP (2018). Photodynamic therapy and diagnosis: principles and comparative aspects. Vet J.

[196] Zhang J, Jiang C, Figueiró Longo JP, Azevedo RB, Zhang H, Muehlmann LA (2018). An updated overview on the development of new photosensitizers for anticancer photodynamic therapy. Acta pharmaceutica Sinica B.

[197] Keene JP, Kessel D, Land EJ, Redmond RW, Truscott TG (1986). Direct detection of singlet oxygen sensitized by haematoporphyrin and related compounds. Photochem Photobiol.

[198] Colombeau L, Acherar S, Baros F, Arnoux P, Gazzali AM, Zaghdoudi K (2016). Inorganic nanoparticles for photodynamic therapy. Top Curr Chem.

[199] Mokoena D, George BP, Abrahamse H (2022). Conjugation of hypericin to gold nanoparticles for enhancement of photodynamic therapy in MCF-7 breast cancer cells. Pharmaceutics.

[200] Zhang Y, Ye Z, He R, Li Y, Xiong B, Yi M (2023). Bovine serum albumin-based and dual-responsive targeted hollow mesoporous silica nanoparticles for breast cancer therapy. Colloids Surf B.

[201] Nosrati H, Abbasi R, Charmi J, Rakhshbahar A, Aliakbarzadeh F, Danafar H (2018). Folic acid conjugated bovine serum albumin: an efficient smart and tumor targeted biomacromolecule for inhibition folate receptor positive cancer cells. Int J Biol Macromol.

[202] Lim DJ (2021). Methylene blue-based nano and microparticles: fabrication and applications in photodynamic therapy. Polymers.

[203] Vankayala R, Sagadevan A, Vijayaraghavan P, Kuo CL, Hwang KC (2011). Metal nanoparticles sensitize the formation of singlet oxygen. Angew Chem Int Ed Engl.

[204] Wu H, Jiang Q, Luo K, Zhu C, Xie M, Wang S (2021). Synthesis of iridium-based nanocomposite with catalase activity for cancer phototherapy. J Nanobiotechnol.

[205] Yuan X, Cen J, Chen X, Jia Z, Zhu X, Huang Y (2022). Iridium oxide nanoparticles mediated enhanced photodynamic therapy combined with photothermal therapy in the treatment of breast cancer. J Colloid Interface Sci.

[206] Deng X, Zhao R, Song Q, Zhang Y, Zhao H, Hu H (2022). Synthesis of dual-stimuli responsive metal organic framework-coated iridium oxide nanocomposite functionalized with tumor targeting albumin-folate for synergistic photodynamic/photothermal cancer therapy. Drug Deliv.

[207] Li L, Yu Y, Ye GJ, Ge Q, Ou X, Wu H (2014). Black phosphorus field-effect transistors. Nat Nanotechnol.

[208] Qin L, Jiang S, He H, Ling G, Zhang P (2020). Functional black phosphorus nanosheets for cancer therapy. J Control Release.

[209] Wang H, Yang X, Shao W, Chen S, Xie J, Zhang X (2015). Ultrathin black phosphorus nanosheets for efficient singlet oxygen generation. J Am Chem Soc.

[210] Redmond RW, Gamlin JN (1999). A compilation of singlet oxygen yields from biologically relevant molecules. Photochem Photobiol.

[211] Mang TS, Allison R, Hewson G, Snider W, Moskowitz R (1998). A phase II/III clinical study of tin ethyl etiopurpurin (Purlytin)-induced photodynamic therapy for the treatment of recurrent cutaneous metastatic breast cancer. Cancer J Sci Am.

[212] Morrison SA, Hill SL, Rogers GS, Graham RA (2014). Efficacy and safety of continuous low-irradiance photodynamic therapy in the treatment of chest wall progression of breast cancer. J Surg Res.

[213] Loboda A, Smolanka I, Orel VE, Syvak L, Golovko T, Dosenko I (2020). Efficacy of combination neoadjuvant chemotherapy and regional inductive moderate hyperthermia in the treatment of patients with locally advanced breast cancer. Technol Cancer Res Treat.

[214] Rahban D, Doostan M, Salimi A (2020). Cancer therapy; prospects for application of nanoparticles for magnetic-based hyperthermia. Cancer Invest.

[215] Gilchrist RK, Medal R, Shorey WD, Hanselman RC, Parrott JC, Taylor CB (1957). Selective inductive heating of lymph nodes. Ann Surg.

[216] Salimi M, Sarkar S, Saber R, Delavari H, Alizadeh AM, Mulder HT (2018). Magnetic hyperthermia of breast cancer cells and MRI relaxometry with dendrimer-coated iron-oxide nanoparticles. Cancer Nanotechnol.

[217] Gao F, Xie W, Miao Y, Wang D, Guo Z, Ghosal A (2019). Magnetic hydrogel with optimally adaptive functions for breast cancer recurrence prevention. Adv Healthc Mater.

[218] Sun R, Chen H, Zheng J, Yoshitomi T, Kawazoe N, Yang Y (2023). Composite scaffolds of gelatin and Fe_3_O_4_ nanoparticles for magnetic hyperthermia-based breast cancer treatment and adipose tissue regeneration. Adv Healthc Mater.

[219] Jones EL, Prosnitz LR, Dewhirst MW, Marcom PK, Hardenbergh PH, Marks LB (2004). Thermochemoradiotherapy improves oxygenation in locally advanced breast cancer. Clin Cancer Res.

[220] Vujaskovic Z, Kim DW, Jones E, Lan L, McCall L, Dewhirst MW (2010). A phase I/II study of neoadjuvant liposomal doxorubicin, paclitaxel, and hyperthermia in locally advanced breast cancer. Int J Hyperth.

[221] Xue W, Liu XL, Ma H, Xie W, Huang S, Wen H (2018). AMF responsive DOX-loaded magnetic microspheres: transmembrane drug release mechanism and multimodality postsurgical treatment of breast cancer. J Mater Chem B.

[222] Wang X, Zhong X, Bai L, Xu J, Gong F, Dong Z (2020). Ultrafine titanium monoxide (TiO_1+x_) nanorods for enhanced sonodynamic therapy. J Am Chem Soc.

[223] Cheng CA, Chen W, Zhang L, Wu HH, Zink JI (2019). A responsive mesoporous silica nanoparticle platform for magnetic resonance imaging-guided high-intensity focused ultrasound-stimulated cargo delivery with controllable location, time, and dose. J Am Chem Soc.

[224] Ouyang J, Tang Z, Farokhzad N, Kong N, Kim NY, Feng C (2020). Ultrasound mediated therapy: recent progress and challenges in nanoscience. Nano Today.

[225] Xu M, Zhou L, Zheng L, Zhou Q, Liu K, Mao Y (2021). Sonodynamic therapy-derived multimodal synergistic cancer therapy. Cancer Lett.

[226] Gong F, Cheng L, Yang N, Betzer O, Feng L, Zhou Q (2019). Ultrasmall oxygen-deficient bimetallic oxide MnWO_X_ nanoparticles for depletion of endogenous GSH and enhanced sonodynamic cancer therapy. Adv Mater.

[227] Sun L, Wang P, Zhang J, Sun Y, Sun S, Xu M (2021). Design and application of inorganic nanoparticles for sonodynamic cancer therapy. Biomater Sci.

[228] Loke YL, Beishenaliev A, Wang PW, Lin CY, Chang CY, Foo YY (2023). ROS-generating alginate-coated gold nanorods as biocompatible nanosonosensitisers for effective sonodynamic therapy of cancer. Ultrason Sonochem.

[229] Geng B, Zhang S, Yang X, Shi W, Li P, Pan D (2022). Cu_2−x_O@TiO_2-y_ Z-scheme heterojunctions for sonodynamic-chemodynamic combined tumor eradication. Chem Eng J.

[230] Cao Y, Wu T, Dai W, Dong H, Zhang X (2019). TiO_2_ nanosheets with the Au nanocrystal-decorated edge for mitochondria-targeting enhanced sonodynamic therapy. Chem Mater.

[231] Deepagan VG, You DG, Um W, Ko H, Kwon S, Choi KY (2016). Long-circulating Au-TiO_2_ nanocomposite as a sonosensitizer for ROS-mediated eradication of cancer. Nano Lett.

[232] Ouyang J, Xie A, Zhou J, Liu R, Wang L, Liu H (2022). Minimally invasive nanomedicine: nanotechnology in photo-/ultrasound-/radiation-/magnetism-mediated therapy and imaging. Chem Soc Rev.

[233] Li Y, Chen W, Kang Y, Zhen X, Zhou Z, Liu C (2023). Nanosensitizer-mediated augmentation of sonodynamic therapy efficacy and antitumor immunity. Nat Commun.

[234] Baeza A, Colilla M, Vallet-Regí M (2015). Advances in mesoporous silica nanoparticles for targeted stimuli-responsive drug delivery. Expert Opin Drug Deliv.

[235] Li T, Geng Y, Zhang H, Wang J, Feng Y, Chen Z (2020). A versatile nanoplatform for synergistic chemo-photothermal therapy and multimodal imaging against breast cancer. Expert Opin Drug Deliv.

[236] Anselmo AC, Mitragotri S (2016). Nanoparticles in the clinic. Bioeng Transl Med.

[237] Shen X, Li T, Chen Z, Xie X, Zhang H, Feng Y (2019). NIR-light-triggered anticancer strategy for dual-modality imaging-guided combination therapy via a bioinspired hybrid PLGA nanoplatform. Mol Pharm.

[238] Skrivanová K, Skorpíková J, Svihálek J, Mornstein V, Janisch R (2006). Photochemical properties of a potential photosensitiser indocyanine green in vitro. J Photochem Photobiol B.

[239] Leiloglou M, Kedrzycki MS, Chalau V, Chiarini N, Thiruchelvam PTR, Hadjiminas DJ (2022). Indocyanine green fluorescence image processing techniques for breast cancer macroscopic demarcation. Sci Rep.

[240] Najafipour A, Gharieh A, Fassihi A, Sadeghi-Aliabadi H, Mahdavian AR (2021). MTX-loaded dual thermoresponsive and pH-responsive magnetic hydrogel nanocomposite particles for combined controlled drug delivery and hyperthermia therapy of cancer. Mol Pharm.

[241] Kang X, Sun T, Zhang L, Zhou C, Xu Z, Du M (2021). Synergistic theranostics of magnetic resonance imaging and photothermal therapy of breast cancer based on the janus nanostructures Fe_3_O_4_-Au_shell_-PEG. Int J Nanomed.

[242] Zeng J, Gong M, Wang D, Li M, Xu W, Li Z (2019). Direct synthesis of water-dispersible magnetic/plasmonic heteronanostructures for multimodality biomedical imaging. Nano Lett.

[243] Xia X, Wang Y, Ruditskiy A, Xia Y (2013). 25th anniversary article: galvanic replacement: a simple and versatile route to hollow nanostructures with tunable and well-controlled properties. Adv Mater.

[244] Ma X, Wang Y, Liu X-L, Ma H, Li G, Li Y (2019). Fe_3_O_4_–Pd Janus nanoparticles with amplified dual-mode hyperthermia and enhanced ROS generation for breast cancer treatment. Nanoscale Horiz.

[245] Estelrich J, Busquets MA (2018). Iron oxide nanoparticles in photothermal therapy. Molecules.

[246] Włodarczyk A, Gorgoń S, Radoń A, Bajdak-Rusinek K (2022). Magnetite nanoparticles in magnetic hyperthermia and cancer therapies: challenges and perspectives. Nanomaterials.

[247] Shi S, Huang Y, Chen X, Weng J, Zheng N (2015). Optimization of surface coating on small Pd nanosheets for in vivo near-infrared photothermal therapy of tumor. ACS Appl Mater Interfaces.

[248] Ranji-Burachaloo H, Gurr PA, Dunstan DE, Qiao GG (2018). Cancer treatment through nanoparticle-facilitated fenton reaction. ACS Nano.

[249] Mo S, Chen X, Chen M, He C, Lu Y, Zheng N (2015). Two-dimensional antibacterial Pd@Ag nanosheets with a synergetic effect of plasmonic heating and Ag^+^ release. J Mater Chem B.

[250] Bhattarai S, Mackeyev Y, Venkatesulu BP, Krishnan S, Singh PK (2021). CXC chemokine receptor 4 (CXCR4) targeted gold nanoparticles potently enhance radiotherapy outcomes in breast cancer. Nanoscale.

[251] Lin HC, Hsu KF, Lai CL, Wu TC, Chen HF, Lai CH (2020). Mannoside-modified branched gold nanoparticles for photothermal therapy to MDA-MB-231 cells. Molecules.

[252] Han R, Xiao Y, Yang Q, Pan M, Hao Y, He X (2021). Ag_2_S nanoparticle-mediated multiple ablations reinvigorates the immune response for enhanced cancer photo-immunotherapy. Biomaterials.

[253] Amoli-Diva M, Sadighi-Bonabi R, Pourghazi K, Hadilou N (2018). Tunable surface plasmon resonance-based remote actuation of bimetallic core-shell nanoparticle-coated stimuli responsive polymer for switchable chemo-photothermal synergistic cancer therapy. J Pharm Sci.

[254] Saharkhiz S, Zarepour A, Zarrabi A (2023). A new theranostic pH-responsive niosome formulation for doxorubicin delivery and bio-imaging against breast cancer. Int J Pharm.

[255] Xu N, Piao M, Arkin K, Ren L, Zhang J, Hao J (2019). Imaging of water soluble CdTe/CdS core-shell quantum dots in inhibiting multidrug resistance of cancer cells. Talanta.

[256] Hu S, Ren Y, Wang Y, Li J, Qu J, Liu L (2019). Surface plasmon resonance enhancement of photoluminescence intensity and bioimaging application of gold nanorod@CdSe/ZnS quantum dots. Beilstein J Nanotechnol.

[257] Hong H, Wang F, Zhang Y, Graves SA, Eddine SB, Yang Y (2015). Red fluorescent zinc oxide nanoparticle: a novel platform for cancer targeting. ACS Appl Mater Interfaces.

[258] Hong EJ, Sivakumar P, Ravichandran V, Choi DG, Kim YS, Shim MS (2019). Pro-oxidant drug-loaded Au/ZnO hybrid nanoparticles for cancer-specific chemo-photodynamic combination therapy. ACS Biomater Sci Eng.

[259] Akbarzadeh M, Babaei M, Abnous K, Taghdisi SM, Peivandi MT, Ramezani M (2019). Hybrid silica-coated Gd-Zn-Cu-In-S/ZnS bimodal quantum dots as an epithelial cell adhesion molecule targeted drug delivery and imaging system. Int J Pharm.

[260] Deng H, Yang Y, Zuo T, Fang T, Xu Y, Yang J (2021). Multifunctional ZnO@CuS nanoparticles cluster synergize chemotherapy and photothermal therapy for tumor metastasis. Nanomedicine.

[261] Ramachandran P, Khor BK, Lee CY, Doong RA, Oon CE, Thanh NTK (2022). N-Doped graphene quantum dots/titanium dioxide nanocomposites: a study of ROS-forming mechanisms, cytotoxicity and photodynamic therapy. Biomedicines.

[262] Ninomiya K, Fukuda A, Ogino C, Shimizu N (2014). Targeted sonocatalytic cancer cell injury using avidin-conjugated titanium dioxide nanoparticles. Ultrason Sonochem.

[263] Zhang X, Wu J, Williams GR, Niu S, Qian Q, Zhu L-M (2019). Functionalized MoS2-nanosheets for targeted drug delivery and chemo-photothermal therapy. Colloids Surf B.

[264] Li X, Gong Y, Zhou X, Jin H, Yan H, Wang S (2016). Facile synthesis of soybean phospholipid-encapsulated MoS2 nanosheets for efficient in vitro and in vivo photothermal regression of breast tumor. Int J Nanomed.

[265] Ramírez-García G, De la Rosa E, López-Luke T, Panikar SS, Salas P (2019). Controlling trapping states on selective theranostic core@shell (NaYF_4_:Yb, Tm@TiO_2_-ZrO_2_) nanocomplexes for enhanced NIR-activated photodynamic therapy against breast cancer cells. Dalton Trans.

[266] Zeng L, Pan Y, Tian Y, Wang X, Ren W, Wang S (2015). Doxorubicin-loaded NaYF4:Yb/Tm-TiO_2_ inorganic photosensitizers for NIR-triggered photodynamic therapy and enhanced chemotherapy in drug-resistant breast cancers. Biomaterials.

[267] Zhou L, Yang T, Wang J, Wang Q, Lv X, Ke H (2017). Size-tunable Gd_2_O_3_@albumin nanoparticles conjugating chlorin e6 for magnetic resonance imaging-guided photo-induced therapy. Theranostics.

[268] Antoniak MA, Pązik R, Bazylińska U, Wiwatowski K, Tomaszewska A, Kulpa-Greszta M (2021). Multimodal polymer encapsulated CdSe/Fe_3_O_4_ nanoplatform with improved biocompatibility for two-photon and temperature stimulated bioapplications. Mater Sci Eng C Mater Biol Appl.

[269] Salimi M, Sarkar S, Hashemi M, Saber R (2020). Treatment of breast cancer-bearing BALB/c mice with magnetic hyperthermia using dendrimer functionalized iron-oxide nanoparticles. Nanomaterials.

[270] Yuan M, Liang S, Yang L, Li F, Liu B, Yang C (2023). Rational design of platinum-bismuth sulfide Schottky heterostructure for sonocatalysis-mediated hydrogen therapy. Adv Mater.

[271] Zhang X, Li Y, Wei M, Liu C, Yu T, Yang J (2019). Cetuximab-modified silica nanoparticle loaded with ICG for tumor-targeted combinational therapy of breast cancer. Drug Deliv.

[272] Cai D, Liu L, Han C, Ma X, Qian J, Zhou J (2019). Cancer cell membrane-coated mesoporous silica loaded with superparamagnetic ferroferric oxide and paclitaxel for the combination of chemo/magnetocaloric therapy on MDA-MB-231 cells. Sci Rep.

[273] de Melo-Diogo D, Costa EC, Alves CG, Lima-Sousa R, Ferreira P, Louro RO (2018). POxylated graphene oxide nanomaterials for combination chemo-phototherapy of breast cancer cells. Eur J Pharm Biopharm.

[274] Melo BL, Lima-Sousa R, Alves CG, Ferreira P, Moreira AF, Correia IJ (2021). Sulfobetaine methacrylate-albumin-coated graphene oxide incorporating IR780 for enhanced breast cancer phototherapy. Nanomedicine.

[275] Jia Y, Weng Z, Wang C, Zhu M, Lu Y, Ding L (2017). Increased chemosensitivity and radiosensitivity of human breast cancer cell lines treated with novel functionalized single-walled carbon nanotubes. Oncol Lett.

[276] McKernan P, Virani NA, Faria GNF, Karch CG, Prada Silvy R, Resasco DE (2021). Targeted single-walled carbon nanotubes for photothermal therapy combined with immune checkpoint inhibition for the treatment of metastatic breast cancer. Nanoscale Res Lett.

[277] Lin Z, Liu Y, Ma X, Hu S, Zhang J, Wu Q (2015). Photothermal ablation of bone metastasis of breast cancer using PEGylated multi-walled carbon nanotubes. Sci Rep.

[278] Medicine NLo. https://clinicaltrials.gov/search?cond=Breast%20Cancer&term=nanoparticles. Accessed 26 May 2024.

